# Integration of whey and mycorrhizal symbiosis: a sustainable biocontrol strategy against *Zucchini yellow mosaic virus* in squash

**DOI:** 10.1007/s00572-026-01262-7

**Published:** 2026-04-24

**Authors:** Gökhan Boyno, Nisa Asel Tatar, Mustafa Usta, Necmettin Teniz, Semra Demir

**Affiliations:** 1 Department of Plant Protection, Faculty of Agriculture, Van Yuzuncu Yil University, Van, 65090 Türkiye; 2 Department of Agricultural Biotechnology, Faculty of Agriculture, Van Yuzuncu Yil University, Van, 65090 Türkiye

**Keywords:** Whey, Arbuscular mycorrhizal fungi (AMF), *Zucchini yellow mosaic virus* (ZYMV), Biocontrol, Induced Resistance

## Abstract

*Zucchini yellow mosaic virus* [*Potyvirus cucurbita flavitesselati*, (ZYMV-Z)] is a major pathogen causing severe yield losses in global Squash (*Cucurbita pepo L*). production, with limited sustainable control strategies available. This study aimed to elucidate the biocontrol potential of whey, a by-product of the dairy industry with recognized biofertilizer and biostimulant properties, in integration with arbuscular mycorrhizal fungi (AMF-M) symbiosis, and to uncover the underlying multilayered defense mechanisms in ZYMV-infected squash plants. ZYMV-infected and non-infected plants were treated with whey (applied-foliar-Wf or via the rhizosphere-Wr) and M, either individually or in combination (WfMZ, WrMZ). Plant growth traits, physiological indicators, biochemical defense responses, soil properties, and mycorrhizal colonization parameters were comprehensively evaluated. ZYMV infection markedly suppressed plant growth, while the integration of whey and AMF significantly mitigated these adverse effects. Notably, the WfMZ combination maintained chlorophyll content and substantially reduced disease severity even under viral stress. This synergistic interaction was associated with a multifaceted activation of the plant’s defense machinery: WfMZ and WrMZ treatments induced the highest total antioxidant activity, phenolic content, proline accumulation, and PAL/CAT enzyme activities. whey acted as a microbial biostimulant by enhancing AMF spore density and root colonization, whereas AMF improved plant-phosphorus uptake and buffered ZYMV-induced rhizosphere acidification. Overall, the integration of whey and AMF represents an effective and sustainable biocontrol strategy against ZYMV. This efficacy stems from the synergy of multiple mechanisms, including direct biostimulation, physiological buffering, rhizosphere engineering, and systemic defense induction along the root–shoot axis. The findings highlight a promising approach for converting agricultural by-products into high-value biocontrol agents for managing viral diseases in plants.

**Supplementary Information:**

The online version contains supplementary material available at 10.1007/s00572-026-01262-7.

## Introduction

Squash (*Cucurbita pepo L.*) is one of the most important vegetable crops worldwide due to its high nutritional value and economic significance. However, viral diseases, particularly *Zucchini yellow mosaic virus* [*Potyvirus cucurbita flavitesselati* (ZYMV)] and other *potyviruses*, are among the major factors responsible for substantial yield and quality losses in production systems (Güller et al. 2024). ZYMV infection is characterized by yellow mosaic patterns, vein banding, leaf malformation, and fruit deformation, which can result in yield losses of up to 50–100% under severe conditions (Güller and Usta 2020). The virus is transmitted both mechanically and by aphid vectors (*Aphis* spp.), facilitating rapid spread and epidemic outbreaks in the field (Gal-On 2007). Since chemical control methods are ineffective against viruses, integrated biological and ecological management strategies have become increasingly important for sustainable disease control.

In modern agriculture, research aimed at enhancing agricultural sustainability has increasingly focused on plant–microbe interactions and the valorization of agricultural by-products. In this context, whey, a by-product of the dairy industry, has gained attention as a low-cost biofertilizer and biostimulant source (Puglia et al. 2021). Due to its rich composition of lactic acid, mineral nutrients, amino acids, and biologically active compounds, whey can improve soil health and activate plant defense mechanisms. In line with this, Erman et al. (2011) reported that whey application positively influenced growth and physiological performance in chickpea. Similarly, Demir et al. (2015) demonstrated that whey enhances soil microbial activity, accelerates organic matter transformation, strengthens plant metabolic processes, and can trigger defense responses under various biotic stress conditions. However, the effects of whey alone are often limited, whereas its co-application with beneficial microorganisms can generate a synergistic response (Demir and Özrenk 2009; Erman et al. 2011; Ekincialp et al. 2016).

At this point, arbuscular mycorrhizal fungi (AMF) have emerged as a key biological tool in sustainable crop protection. AMF establish a symbiotic association with plant roots by forming arbuscules and vesicles within the root cortex, thereby enhancing the uptake of mineral nutrients—particularly phosphorus—and improving water use efficiency (Boyno et al. 2022). More importantly, AMF colonization strengthens plant defense responses by inducing systemic resistance (ISR) and modulating hormonal signaling networks, as extensively reviewed in recent studies (Bedini et al. 2018; Shi et al. 2023). Recent evidence suggests that AMF play a crucial role in mitigating virus-induced stress by maintaining reactive oxygen species (ROS) homeostasis, enhancing antioxidant enzyme activities, and reinforcing cell wall structures, as highlighted in recent comprehensive reviews (Bedini et al. 2018; Zou et al. 2021; Boyno et al. 2025a; Fiorilli et al. 2024).

Both individual and combined applications of whey and AMF have been reported to enhance plant resistance against various pathogens across different crops (Demir and Özrenk 2009; Erman et al. 2011; Ekincialp et al. 2016; Boyno et al. 2025a). However, their effects on viral pathogens remain poorly understood. Some studies have even reported that the effects of AMF alone against viruses can be limited or, in certain cases, may exacerbate viral infection (Sipahioğlu et al. 2009; Demir and Sipahioğlu 2015; Miozzi et al. 2019). In particular, the mechanisms underlying the synergistic interaction between whey and AMF in modulating plant defense responses against viral infections have not yet been elucidated. This study investigates the potential defense mechanisms triggered by whey, applied either through foliar or rhizospheric routes, in combination with AMF against ZYMV infection in squash plants. The central hypothesis posits that the bioactivecomprising carbon- and nitrogen-based compounds, may promote AMF root colonization and functionality, thereby enhancing plant metabolic and antioxidative defense systems. Conversely, the symbiotic structures formed by AMF could facilitate the translocation of whey-induced metabolites within plant tissues, thereby restricting viral replication. Overall, this research aims to evaluate how whey–AMF interactions influence plant growth, physiological performance, antioxidant enzyme activity, and soil biochemical properties under ZYMV stress. The outcomes are expected to provide new insights into the development of eco-friendly, low-cost, and sustainable strategies for viral disease management in environmentally responsible crop production systems.

## Materials and methods

### Materials and experimental conditions

In this study, Squash (*Cucurbita pepo L.* cv. ‘Sakız’), an economically important and widely cultivated crop known to be susceptible to *Zucchini yellow mosaic virus* (ZYMV, *Potyvirus cucurbita flavitesselati*), was used as the test plant (Güller and Usta 2019). The seeds were obtained from a certified agricultural supplier (Bursa Seed Corporation, Bursa, Türkiye). The test pathogen was ZYMV, while the beneficial microorganism used was *Funneliformis mosseae* (T.H. Nicolson and Gerd.) C. Walker and A. Schüßler (BEG12). Both microbial materials were supplied by the Phytopathology Laboratory of Van Yüzüncü Yıl University (Türkiye). As an organic amendment, whey was used, provided by the Department of Food Engineering at Van Yüzüncü Yıl University. The basic properties of the whey are presented in Table 1.

**Table 1:** Composition of the whey used in the study

Component	Amount
Dry matter (%)	7.85
Fat (%)	0.50
Acidity (% lactic acid)	0.11
pH	6.74
Protein (% of dry matter)	10.72
Ash (%)	0.50

The plant growth medium consisted of a mixture of soil, sterilized pumice, and sand in a 2:2:1 (v/v/v) ratio. Both the soil and sand were sieved through a 2 mm mesh and subsequently sterilized in an autoclave at 121 °C for 1 h to eliminate microbial contamination. Prior to the experiment, sterilized soil samples were examined under a microscope after ultrasonic centrifugation to confirm the absence of AMF (Boyno et al. 2023), and no spores were detected. In addition, after root cleaning and staining, no mycorrhizal structures were observed in root samples taken from randomly selected uninoculated [M(–)] plants. All experiments were conducted in the climate chamber of the Faculty of Agriculture, Van Yüzüncü Yıl University (38°34′03″ N, 43°16′50″ E). During the experiment, the temperature was maintained at 24 ± 2 °C and the relative humidity at approximately 60 ± 3%. The light conditions were supplemented with fluorescent lamps in addition to natural illumination, resulting in a 16 h light/8 h dark photoperiod.

### Experimental design and treatments

The experiment was conducted in a controlled climate chamber under standardized environmental conditions (25 ± 2 °C, 16 h light/8 h dark photoperiod, relative humidity 60–70%). The study was performed once using a completely randomized factorial design. Each treatment consisted of five biological replicates (one plant per pot). Sterile plastic pots (3 L capacity; 16 × 18 cm) were used as experimental units. Prior to use, pots were disinfected by immersion in a 2% sodium hydroxide (NaOH) solution for 5 min and thoroughly rinsed. The *Funneliformis mosseae* inoculum was mixed homogeneously into the growth substrate at a rate of 10% (v/v), corresponding to approximately 160–240 spores g⁻¹ soil. Non-mycorrhizal control pots received an equal amount of sterilized sand to maintain comparable substrate conditions.

Before sowing, squash (*Cucurbita pepo* L.) seeds were surface-sterilized in a 1% sodium hypochlorite (NaOCl) solution for 3 min, followed by rinsing with sterile distilled water. At the cotyledon stage, plants were mechanically inoculated with *Zucchini yellow mosaic virus* (ZYMV). Briefly, cotyledons were lightly dusted with carborundum (600 mesh) as an abrasive, and viral inoculum prepared from ZYMV-infected leaf tissue homogenized in phosphate buffer (0.01 M, pH 7.0) was gently rubbed onto the leaf surface. After inoculation, leaves were rinsed with distilled water to remove excess inoculum and abrasive particles. From the appearance of the first true leaves onward, whey was applied at a rate of 50 mL kg⁻¹ (Özrenk et al. 2003). Two application methods were evaluated independently: (i) foliar spray and (ii) rhizospheric application via irrigation. All treatment combinations are listed in Table S1.

The experiment was terminated eight weeks after sowing. Growth (biometric, physiological, and biochemical), mycorrhizal, soil parameters, and disease severity were assessed. The full names and units of all measured variables are provided in Table S2. For measurements requiring subsampling (e.g., physiological, biochemical, mycorrhizal, and soil parameters), three technical replicates per plant were analyzed and averaged prior to statistical evaluation. A comprehensive description of all methodological procedures is provided in Table S3.

### Assessment of plant biometric parameters

At the end of the experiment, plant growth parameters (shoot/root fresh and dry weights, shoot/root length, and stem diameter) were measured using standard procedures. Fresh weights were recorded immediately after harvesting, and dry weights were obtained after oven-drying at 70 °C. Stem diameter was measured with a digital caliper.

### Assessment of Mycorrhizal Parameters

Mycorrhizal colonization in squash roots was assessed following the method of Phillips and Hayman (1970), with minor modifications as described by Boyno (2024). Root samples were cleaned in KOH, acidified, and stained with lactophenol blue.

**Mycorrhizal frequency (%F)** was calculated as the ratio of colonized root fragments to the total number of observed fragments. **Mycorrhizal intensity (%M)** was assessed using the 0–5 scale proposed by Trouvelot et al. (1986), where: 0 = no propagules; 1 = < 10%; 2 = 10–30%; 3 = 30–50%; 4 = 50–90%; and 5 = 90–100% presence of propagules. The mycorrhizal intensity was calculated according to the following equation:

$${\%}\mathrm{M}=[(95{\times}\mathrm{n}5)+(70{\times}n4)+(30{\times}\mathrm{n}3)+(5{\times}\mathrm{n}2)+(1{\times}\mathrm{n}1)]\div\mathrm{N}$$

Where:

*n₁–n₅* represents the number of roots in each class.

*N* = the total number of observed root segments.

Arbuscular mycorrhizal spore density in the rhizosphere was quantified using the ultrasonic centrifugation technique of Boyno et al. (2023). The total number of AMF spores per gram of soil was calculated according to the following formula (Boyno et al. 2023):

$$\mathrm{TSN}=[\mathrm{SN}{\times}\mathrm{W}]\div\mathrm{S}$$

where:

TSN = total number of AMF spores per gram of rhizosphere soil.

SN = number of AMF spores in 1 mL of suspension.

W = total suspension volume (mL).

S = weight of soil used (g).

### Assessment of Plant Biochemical Activity

Leaf extracts were prepared from fresh tissue, and total phenolic content (TPC), total antioxidant activity (TAA), and proline content were determined following the established methods of Swain and Hillis (1959), Benzie and Strain (1996), and Bates et al. (1973), respectively.

### Assessment of Antioxidant and Defense-Related Enzyme Activities

Catalase (CAT) and phenylalanine ammonia-lyase (PAL) activities were quantified using spectrophotometric methods described by Jebara et al. (2010) and Bhattacharyya and Ward (1988), with minor modifications.

### Assessment of Total Phosphorus and Chlorophyll Content in Plants

Total phosphorus was determined using the vanadomolybdophosphoric acid method (Barton 1948). The total chlorophyll content was determined using a SPAD meter (502-Plus, Konica Minolta, Japan). Measurements were taken from three fully expanded leaves per plant to represent each plant’s overall chlorophyll content (Fischer 2001).

### Assessment of Soil Parameters

Soil organic matter was determined using the potassium dichromate oxidation method described by Nelson and Sommers (1982). Total lime content was measured following Gülçur (1974), based on the reaction of soil CaCO₃ with HCl and the quantification of released CO₂. Soil moisture content was determined gravimetrically according to Schmugge et al. (1980) by oven-drying samples at 105 °C to constant weight. Soil pH and EC were measured using the 1:2.5 soil–water suspension method of Jackson (1958).

### Disease Severity and Molecular Confirmation

Following whey treatments, disease severity was assessed weekly (six times in total). Disease severity (DS) caused by *Zucchini yellow mosaic virus* (ZYMV) was rated using a 0–5 scale: 0 = no symptoms; 1 = < 50% leaf mottling; 2 = > 50% leaf mottling; 3 = mottling and mosaic; 4 = mosaic and leaf deformation; 5 = severe symptoms including filiform leaves (Xu et al. 2004). The percentage of disease severity was calculated using the Townsend Heubergeb formula (Townsend and Heubergeb 1943):

$${\%}\:\mathrm{DS}=[(\mathrm{Scale}\:\mathrm{value}\:{\times}\:\mathrm{Number}\:\mathrm{leaves}\:\mathrm{at}\:\mathrm{that}\:\mathrm{scale})\:{\div}\:\mathrm{Total}\:\mathrm{number}\:\mathrm{of}\:\mathrm{leaves}]\times100$$

Total nucleic acids were extracted using the silica-based method of Foissac et al. (2001). ZYMV infection was detected by reverse transcription polymerase chain reaction (RT-PCR) using specific primers described by Özer et al. (2012) (Table S4). Amplified products were analyzed by agarose gel electrophoresis to confirm viral presence.

### Data Statistics

All data were analyzed using a three-way analysis of variance (ANOVA) in SPSS software (version 21.0; SPSS Inc. Chicago, IL, USA) to evaluate the main effects and interactions of Disease [Z(+)/Z(–)], Mycorrhiza [M(+)/M(–)], and whey [W(–)/Wr/Wf] treatments (Table S5). Comparisons among treatment means were performed using Duncan’s Multiple Range Test (DMRT) at significance levels of *p* < 0.05. All measured variables were tested for normality and transformed when necessary (√x, logx, or the Box-Cox method). To reduce the dataset’s dimensionality and interpret relationships among variables, Principal Component Analysis (PCA) was conducted using XLSTAT (version 2022.3.1, Microsoft Excel) (Notes S1). Additionally, a heat map was generated through ClustVis analysis to visualize data variation and assess patterns and relationships among treatments. Correlation analysis was performed to explore associations among variables and identify both positive and negative trends. All results are presented as “mean ± standard deviation.”

## Results

### Effects of treatments on plant biometric parameters under ZYMV stress

**Fig. 1:**
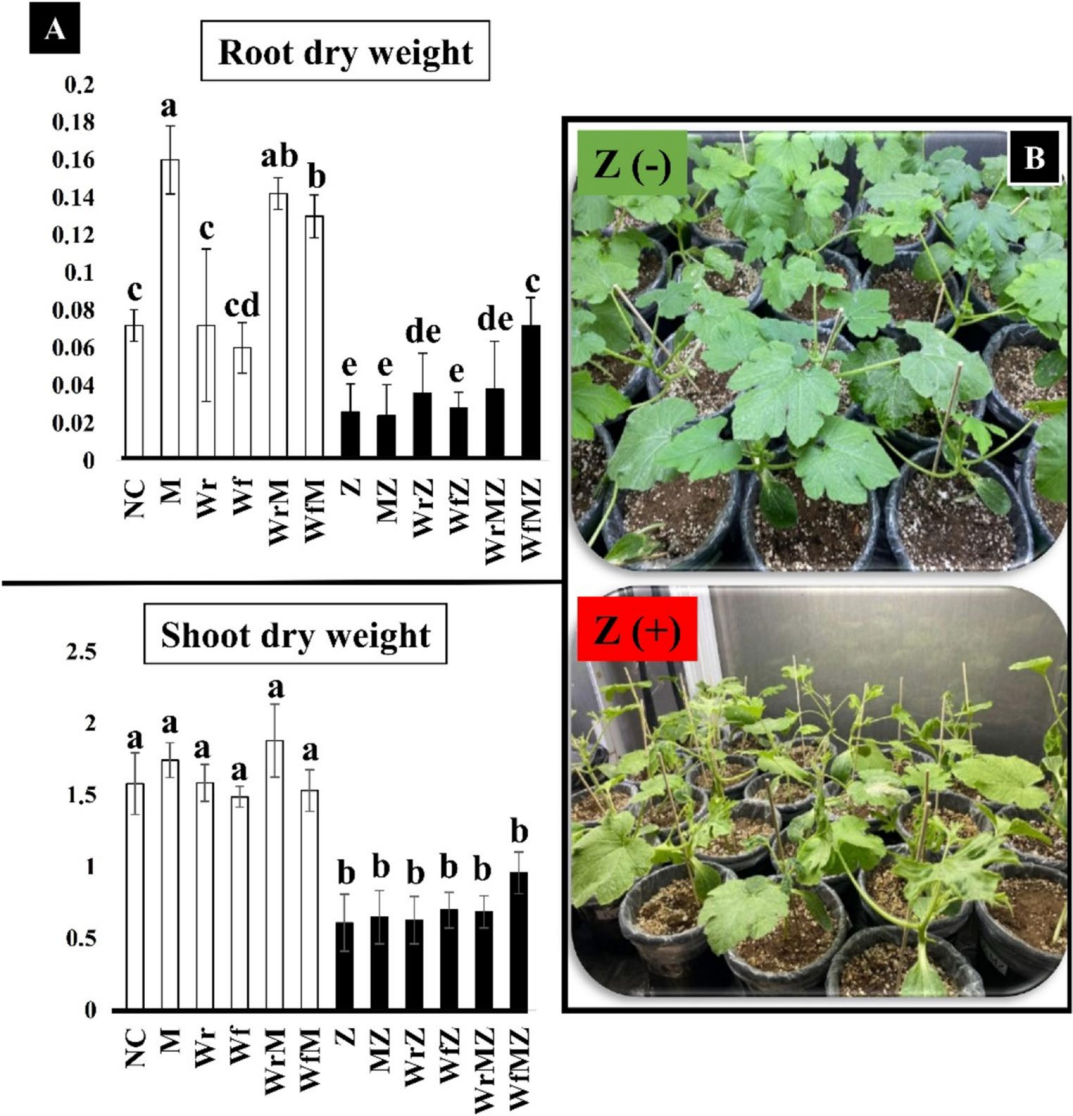
Effects of whey (W) and AMF (M) applications on squash plants under healthy [Z(–)] and ZYMV-infected [Z(+)] conditions. **(A)** Bar graphs showing shoot dry weight (g) and root dry weight (g) under different treatment combinations. Parameters including stem diameter (mm), shoot length (cm), root length (cm), shoot fresh weight (g), and root fresh weight (g) are provided in Fig. S1. White bars represent Z(–) plants and black bars represent Z(+) plants. Data are presented as mean ± SD. Different lowercase letters indicate statistically significant differences among treatments, as determined by Duncan’s multiple-range test (*p* < 0.05). M(+) indicates mycorrhizal plants and M(–) indicates non-mycorrhizal plants. Wf: foliar whey; Wr: rhizospheric whey; NC: negative control. (**B**) (BRepresentative images of squash plants from selected treatment groups under ZYMV-infected [Z(+)] conditions (Z, WfZ, WfMZ) and non-infected [Z(–)] conditions (Wf, WfM). All photographs were taken at the same developmental stage and at the same time point (final assessment)

Under healthy [Z(–)] conditions, both AMF inoculation and whey applications resulted in increases in growth-related parameters compared to the non-treated control (Fig. 1 and Fig. S1). Mycorrhizal plants [M(+)] generally showed higher biomass values than non-mycorrhizal plants [M(–)]. Among whey treatments, rhizospheric application (Wr) was more closely associated with increases in root-related parameters, whereas foliar application (Wf) was mainly associated with shoot elongation. Under ZYMV infection [Z(+)], all biometric parameters decreased relative to Z(–) plants. However, differences among treatments persisted. AMF-inoculated plants maintained higher biomass compared to non-mycorrhizal infected plants. Combined treatments (WrMZ and WfMZ) showed higher values for biomass-related parameters than the ZYMV-infected control, suggesting a positive interaction between AMF and whey under disease conditions. Three-way ANOVA indicated significant main effects of ZYMV infection and AMF inoculation on most growth parameters, whereas interaction effects varied across parameters (Table S5).

Detailed explanations of all abbreviations and their units are provided in Tables S1 and S2. The factorial effects (Z, W, M) and their interactions are presented in detail in Table S5.

### **Effects of Treatments on Mycorrhizal Parameters Under ZYMV Stress**

**Fig. 2:**
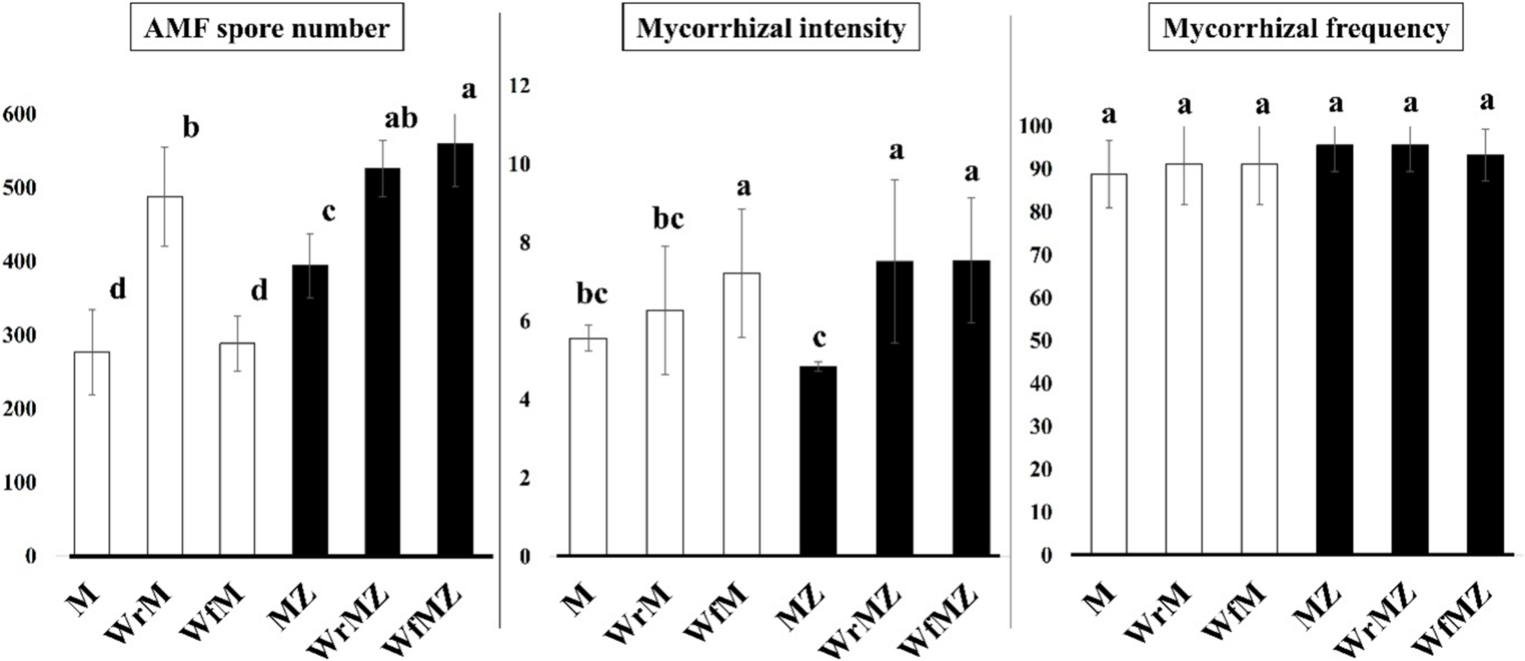
Effects of whey (W) application methods and AMF inoculation (M) on mycorrhizal parameters of squash under healthy [Z(–)] and ZYMV-infected [Z(+)] conditions. Bar graphs showing AMF spore number in rhizosphere soil (spores g⁻¹ soil), mycorrhizal intensity (%M), and mycorrhizal frequency (%F) in roots under different treatment combinations. White bars represent healthy plants [Z(–)], whereas black bars represent ZYMV-infected plants [Z(+)]. Data are presented as mean ± SD. Different lowercase letters above the bars indicate statistically significant differences among treatments, as determined by Duncan’s multiple-range test (*p* < 0.05). Treatment abbreviations: M(+) = mycorrhizal plants; M(–) = non-mycorrhizal plants; Wf = foliar whey application; Wr = rhizospheric whey application; Z = ZYMV infection

The three-way ANOVA revealed significant main effects of AMF inoculation and ZYMV infection on spore number and mycorrhizal intensity, whereas whey application was mainly associated with spore production through interaction effects (Table S5). Under both healthy [Z(–)] and infected [Z(+)] conditions, the combined treatments (WfMZ and WrMZ) showed higher AMF spore numbers compared to AMF alone (MZ) (Fig. 2). Among these, rhizospheric whey (WrMZ) was associated with the highest spore numbers. Mycorrhizal intensity (root colonization) was higher in WfM, WfMZ, and WrMZ treatments compared to AMF-only treatments, indicating a potential association between whey application and intraradical fungal development. In contrast, mycorrhizal frequency remained consistently high (88–96%) across all AMF-inoculated treatments and was not significantly affected by either whey or viral infection, suggesting stable colonization establishment across treatments. Under ZYMV infection, AMF spore numbers in MZ treatments were higher than those observed under healthy conditions, indicating that fungal sporulation varied depending on plant health status. The higher values observed in WrMZ and WfMZ treatments may suggest that whey application influences AMF responses under viral stress conditions (Fig. 2). However, the underlying mechanisms of this response require further investigation.

Detailed explanations of all abbreviations and their units are provided in Tables S1 and S2. The factorial effects (Z, W, M) and their interactions are presented in detail in Table S5.

### Effects of Treatments on Plant Biochemistry, Antioxidant Defense, and Soil Properties under ZYMV Stress

The effects of ZYMV infection, AMF inoculation, and whey application on biochemical parameters, total phosphorus and chlorophyll content in squash plants, as well as on rhizosphere soil properties, varied among treatments (Table 2). Under ZYMV infection [Z(+)], total antioxidant activity, total phenolic content, and proline accumulation were higher than in healthy plants [Z(–)] (Table S5). Among treatments, WrMZ and WfMZ showed higher values for these non-enzymatic antioxidant parameters compared to other treatments. These patterns may suggest that combined applications of whey and AMF are associated with changes in non-enzymatic antioxidant responses under viral stress. PAL and CAT enzyme activities were increased under ZYMV infection alone. In the presence of whey and AMF, variations in enzyme activities were observed, indicating that these factors may influence enzymatic antioxidant responses in addition to virus-induced effects. When grouped according to mycorrhizal status, M(+) plants showed higher total antioxidant activity, phenolic content, and proline levels than M(–) plants (Table S5). In contrast, CAT and PAL activities appeared to be more closely associated with viral infection than with AMF alone. Overall, combined treatments (particularly WrMZ and WfMZ) were associated with higher values across several biochemical parameters. However, the extent to which these responses are directly linked to synergistic effects between whey and AMF remains to be investigated.

**Table 2:** Combined effects of whey (W) and arbuscular mycorrhizal fungi (AMF; M) on biochemical traits, antioxidant defense activities, and selected soil properties of squash plants under healthy [Z(–)] and ZYMV-infected [Z(+)] conditions*

hbT	TAC	TPC	Proline	PAL	CAT	*P*
NC	0.99 ± 0.1 g	1.20 ± 0.1 g	3.73 ± 0.7 g	2.93 ± 0.4 h	5.48 ± 0.2j	2.86 ± 0.2e
M	1.25 ± 0.1f	1.49 ± 0.1f	5.05 ± 0.3f	3.99 ± 0.2 g	8.03 ± 0.2i	3.63 ± 0.3bc
Wr	1.69 ± 0.2e	1.90 ± 0.1e	5.12 ± 0.2f	4.22 ± 0.3 g	8.81 ± 0.2 h	2.97 ± 0.1e
Wf	1.50 ± 0.1e	1.80 ± 0.1e	4.63 ± 0.4f	3.65 ± 0.9gh	9.59 ± 0.2 g	2.83 ± 0.1e
WrM	1.96 ± 0.1d	2.22 ± 0.1d	6.68 ± 0.3e	5.58 ± 0.3f	9.81 ± 0.2 g	3.96 ± 0.1a
WfM	2.22 ± 0.1c	2.61 ± 0.1c	8.12 ± 0.2d	7.10 ± 0.2e	10.59 ± 0.2f	3.66 ± 0.1b
Z	2.65 ± 0.1b	3.65 ± 0.5ab	17.85 ± 0.4bc	15.50 ± 1.1a	13.93 ± 0.3a	2.10 ± 0.1 g
MZ	2.55 ± 0.1b	3.44 ± 0.2b	17.26 ± 0.7c	11.91 ± 0.5d	11.59 ± 0.2d	3.29 ± 0.2d
WrZ	2.86 ± 0.1a	3.65 ± 0.2ab	18.09 ± 0.2b	14.26 ± 1.2bc	12.31 ± 0.2c	2.69 ± 0.2e
WfZ	2.96 ± 0.1a	3.76 ± 0.1a	17.91 ± 0.8b	14.86 ± 1.5ab	13.11 ± 0.2b	2.40 ± 0.1f
WrMZ	2.92 ± 0.4a	3.87 ± 0.5a	19.05 ± 0.6a	13.93 ± 0.4bc	12.11 ± 0.2c	3.38 ± 0.5 cd
WfMZ	3.07 ± 0.8a	3.84 ± 0.1a	18.23 ± 0.2b	13.45 ± 0.1c	11.11 ± 0.2e	3.74 ± 0.1ab
hbT	pH	EC	Moisture	CaCO₃	OM	
NC	7.83 ± 0.1a	153.67 ± 29.0bc	1.35 ± 0.3de	8.53 ± 2.6bc	3.23 ± 0.8 cd	
M	7.80 ± 0.1a	136.00 ± 16.5c	1.75 ± 0.3abc	8.97 ± 1.9bc	3.12 ± 0.7 cd	
Wr	7.76 ± 0.1ab	189.00 ± 8.5a	1.94 ± 0.2ab	9.55 ± 2.0bc	3.86 ± 0.7abc	
Wf	7.81 ± 0.1a	162.33 ± 11.7abc	1.33 ± 0.1e	7.29 ± 2.3c	2.92 ± 0.7 cd	
WrM	7.76 ± 0.1ab	169.00 ± 8.5ab	1.89 ± 0.1ab	13.37 ± 1.9a	4.69 ± 0.5ab	
WfM	7.84 ± 0.1a	162.33 ± 22.5abc	1.36 ± 0.1de	8.91 ± 1.6bc	2.40 ± 0.7d	
Z	7.59 ± 0.1b	152.67 ± 18.6bc	1.66 ± 0.2bcd	8.53 ± 2.6bc	3.90 ± 0.6abc	
MZ	7.89 ± 0.1a	145.00 ± 15.0bc	1.65 ± 0.1bcd	11.42 ± 2.6ab	3.46 ± 0.6bcd	
WrZ	7.72 ± 0.1ab	173.00 ± 11.5ab	2.06 ± 0.1a	8.72 ± 1.1bc	5.28 ± 0.1a	
WfZ	7.58 ± 0.2b	144.33 ± 24.0bc	1.25 ± 0.2e	9.08 ± 1.1bc	4.93 ± 0.1a	
WrMZ	7.76 ± 0.1ab	169.00 ± 8.5ab	1.89 ± 0.1ab	8.60 ± 1.2bc	4.10 ± 0.2ab	
WfMZ	7.71 ± 0.1ab	131.00 ± 3.6c	1.53 ± 0.0cde	9.10 ± 1.2bc	3.85 ± 0.2abc	

*: This table summarizes the effects of whey (W) and arbuscular mycorrhizal fungi (AMF; M) applications on biochemical parameters (total antioxidant activity (TAC), total phenolic content (TPC), and proline), antioxidant enzyme activities (PAL and CAT), and selected soil properties (phosphorus (P), pH, electrical conductivity (EC), moisture content, CaCO₃, and organic matter (OM)) under both healthy [Z(–)] and ZYMV-infected [Z(+)] conditions. Data are presented as mean ± standard deviation (SD). Different lowercase letters within each column indicate statistically significant differences among treatment means according to Duncan’s multiple-range test (*p* < 0.05). Treatment abbreviations: T= Treatments, M(+) = mycorrhizal plants; M(–) = non-mycorrhizal plants; Wf = foliar whey application; Wr = rhizospheric whey application; Z = ZYMV infection; NC: negative control. Detailed explanations of all abbreviations and measurement units are provided in Tables S1 and S2. The factorial effects (Z, W, M) and their interactions are presented in detail in Table S5

Total chlorophyll content was generally higher under non-infected [Z(–)] conditions and showed relatively higher values in the WfMZ treatment under ZYMV infection [Z(+)] (Table S5). Total phosphorus content was highest in the WrM treatment, whereas the lowest values were observed in plants exposed only to ZYMV infection [Z(+)] (Table 2). No significant differences were observed between whey application methods in chlorophyll content, whereas both methods resulted in higher phosphorus levels than non-treated conditions. In general, chlorophyll and phosphorus contents were higher in mycorrhizal [M(+)] plants than in non-mycorrhizal [M(–)] plants, and in Z(–) plants compared to Z(+) plants (Table S5).

Soil parameters also varied among treatments (Table 2). Soil pH was lower in the Z and WfZ treatments than in the other treatments, while differences among the remaining treatments were limited. Electrical conductivity (EC) was highest under the Wr treatment. Soil moisture content was highest in WrZ, followed by Wr, WrM, and WrMZ. The highest CaCO₃ content was observed in the WrM treatment, whereas other treatments showed comparatively lower values. Soil organic matter was higher in WrZ and WfZ treatments, followed by WrM and WrMZ. When considering application strategies, Wr treatments were generally associated with higher EC, soil moisture, and organic matter values, whereas AMF inoculation appeared to be associated with variation in pH and CaCO₃. Soil pH values tended to be lower under ZYMV infection, while other soil parameters showed limited or treatment-dependent variation. Three-way ANOVA indicated that chlorophyll content, total phosphorus, and several soil parameters were significantly affected by treatment factors and/or their interactions (Table S5).

### Effects of treatments on disease severity

Disease severity differed significantly among treatments and their interactions (Table S5; Fig. S2). Disease progression increased over time in all inoculated groups. At the final evaluation, the WfMZ treatment showed the lowest mean disease severity (66%), whereas the other treatments ranged from 72% to 76%. When the main factors were considered, the whey application method was associated with variation in disease severity, with Wf-based treatments generally showing lower values compared to several other treatment combinations. However, no statistically significant main effect of mycorrhizal inoculation [M(+)] was detected compared with non-mycorrhizal [M(–)] plants. Overall, variation in disease severity appeared to be influenced by specific treatment combinations and temporal progression rather than by a single factor alone (Fig. S2).

## Multivariate analyses

### Heat map output

**Fig. 3:**
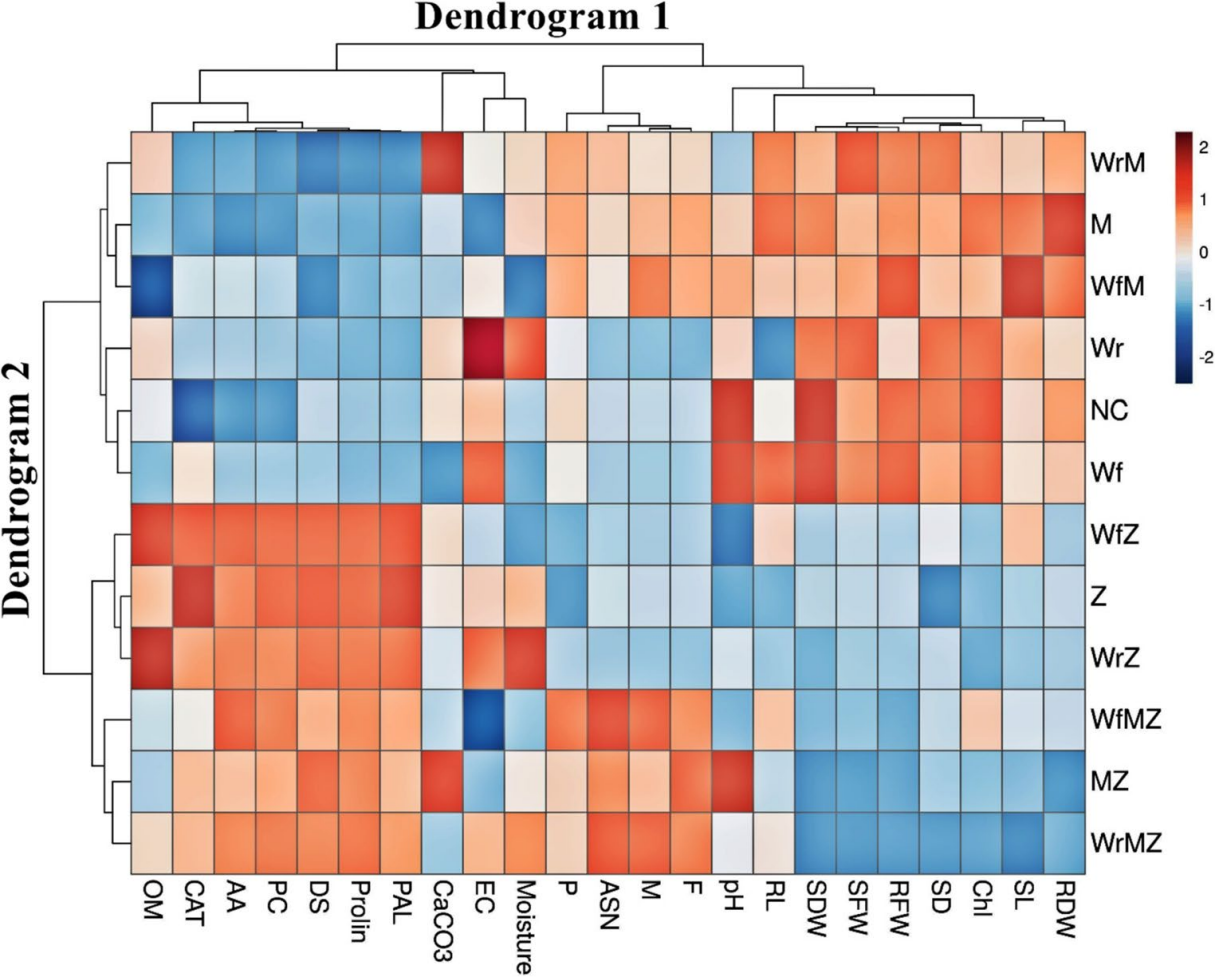
Hierarchical cluster heat map showing the relationships between dependent variables of Squash plants (Dendrogram 1) and different whey and AMF treatments (Dendrogram 2). The color scale represents the relative value of each parameter under the respective treatment, with brown tones indicating higher values (or positive associations) and blue tones indicating lower values (or negative associations). Definitions and units of abbreviations are given in Tables S1 and S2

The heat map illustrating the relationships among dependent variables in squash plants (Dendrogram 1) and treatment groups (Dendrogram 2) is presented in Fig. 3. The clustering pattern showed that treatments were broadly separated into two groups corresponding to the presence [Z(+)] or absence [Z(–)] of disease. Under Z(–) conditions, treatments were generally associated with higher values of plant growth parameters, whereas biochemical and antioxidant parameters tended to show comparatively lower values. Under ZYMV infection [Z(+)], an opposite pattern was observed, with biochemical and antioxidant parameters showing relatively higher values, while growth-related parameters were generally lower. For soil-related variables, variation among treatments was observed, though no consistent pattern emerged across all parameters. A positive association between plant phosphorus (P) content and mycorrhizal treatments was observed in some treatment groups. In terms of mycorrhizal parameters, treatments such as WrMZ, MZ, and WfMZ were grouped together in the clustering analysis, whereas WrM, M, and WfM formed a separate cluster, indicating differences in their overall response patterns (Fig. 3).

### Principal component analysis (PCA)

**Fig. 4:**
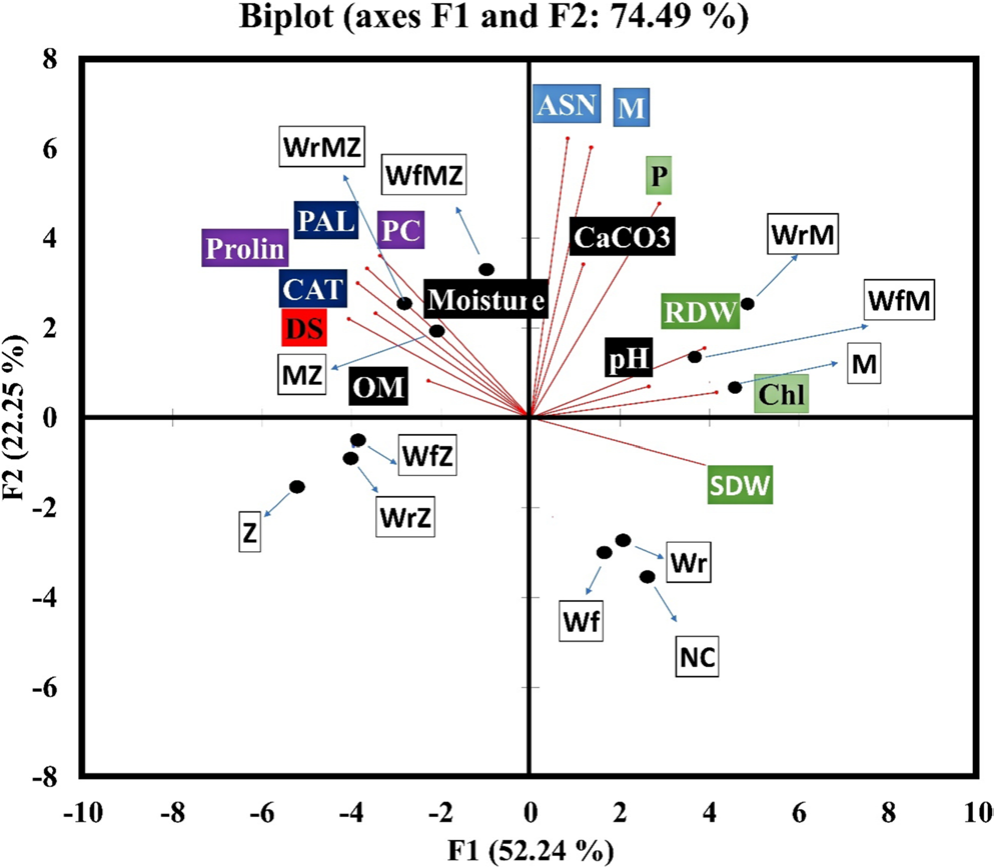
Principal Component Analysis (PCA) biplot illustrating the relationships among biometric, physiological, biochemical, and soil parameters measured in Squash plants under healthy (Z–) and ZYMV-infected (Z+) conditions following different whey and AMF treatments. The analysis explains 74.49% of the total variance (F1 axis: 52.24%; F2 axis: 22.25%). Blue arrows represent the observations (treatments), while labeled red vectors indicate the variables (parameters). Definitions and units of abbreviations are given in Tables S1 and S2

PCA was conducted to examine the relationships among different treatments and the measured variables. The biplot analysis revealed two principal components (F1 and F2) that together explained 74.49% of the total variance. The first principal component (F1) accounted for 52.24% of the total variance, while the second component (F2) explained 22.25% (Fig. 4). The F1 axis clearly distinguished the main stress factor in the study, namely the effect of ZYMV infection. On the negative (left) side of the F1 axis, all ZYMV-infected treatment groups (Z, WrZ, WfZ, MZ, WrMZ, WfMZ) were clustered together, showing a strong positive correlation with disease severity (DS) and biochemical defense parameters, including proline, PAL, CAT, and phenolic compounds (PC) (Fig. 4).

Conversely, on the positive (right) side of the F1 axis, the healthy groups without ZYMV infection (NC, Wr, Wf, M, WrM, WfM) were located, exhibiting a strong positive association with plant growth and morphological parameters such as shoot dry weight (SDW), root dry weight (RDW), and chlorophyll content (Chl) (Fig. 4). The F2 axis separated the effect of mycorrhizal inoculation (AMF) and its relationship with nutrient uptake. The positive (upper) side of the F2 axis comprised all AMF-inoculated treatment groups (M, WrM, WfM, MZ, WrMZ, WfMZ), which showed a strong positive correlation with plant phosphorus (P) content and soil calcium carbonate (CaCO₃) levels. In contrast, the negative (lower) side of the F2 axis included all non-mycorrhizal control groups (NC, Z, Wr, Wf, WrZ, WfZ) (Fig. 4).

### Correlation analysis

Pearson correlation analysis was performed to evaluate the relationships among variables (Fig. S3). The analysis indicated the presence of two broadly contrasting groups of parameters. The first group included plant growth-related parameters, such as stem diameter (SD), stem length (SL), root length (RL), stem fresh weight (SFW), root fresh weight (RFW), stem dry weight (SDW), and root dry weight (RDW), which showed strong positive correlations with each other (*r* > 0.8). The second group comprised biochemical and stress-related parameters, including total antioxidant activity (AA), total phenolic content (PC), proline, PAL, and CAT activity, all of which were positively correlated within this group. In general, negative correlations were observed between growth-related parameters and biochemical stress-related variables. Disease severity (DS) was negatively correlated with several plant growth parameters and positively correlated with biochemical defense-related variables. Total chlorophyll content (Chl) and plant phosphorus (P) content showed positive associations with growth-related parameters, whereas they tended to show negative associations with biochemical parameters and disease severity. Mycorrhizal parameters (M, F) and soil calcium carbonate (CaCO₃) content showed positive correlations with plant phosphorus (P). In addition, mycorrhizal parameters were negatively associated with disease severity (DS) (Fig. S3).

## Discussion

This study comprehensively evaluated the biometric, physiological, biochemical, and symbiotic responses induced by single or combined treatment with whey and arbuscular mycorrhizal fungi (AMF) in Squash plants against *Zucchini yellow mosaic virus* [*Potyvirus cucurbita flavitesselati*, (ZYMV)] infection. The findings indicate that both biostimulant sources possess growth-regulating and defense-inducing effects, and when applied together, they generate a synergistic interaction within the plant–microorganism–pathogen triad.

One of the key findings of our study is that whey and AMF applications enhance plant growth in the absence of stress (Fig. 1). Under ZYMV infection, however, this biostimulant effect shifted toward a protective role. This can be explained by the fact that mycorrhizal fungi provide direct nutrients (amino acids, organic acids) and growth-regulating metabolites (Caballero et al. 2020), while AMF optimize water and mineral uptake (particularly phosphorus) by expanding root surface area (Boyno et al. 2025a).

The basis of the synergy observed in this study lies in the fact that whey and AMF not only exert positive effects on the plant but also mutually enhance each other. Even under ZYMV stress, whey applications (WfMZ and WrMZ) significantly increased both AMF spore density in the rhizosphere and mycorrhizal colonization rates in the roots (Fig. 2). This indicates that whey functions as a microbial biostimulant (Erman et al. 2011; Demir et al. 2015; Malos et al. 2025). The rich organic carbon, amino acids, and soluble compounds in whey created a favorable nutrient environment for AMF hyphal growth and sporulation in the rhizosphere (Demir and Ozrenk 2009; Demir et al. 2015). Whey effectively acted as a “fuel” for the symbiosis, enhancing the efficiency of the mycorrhizal network. According to Ocak and Demir (2012), this suggests that whey can serve as a valuable tool in rhizosphere engineering and may be used to improve the effectiveness of mycorrhizal inoculants. The support provided by whey allowed for the maximization of AMF-mediated benefits, including phosphorus uptake, water optimization, and ISR (Induced Systemic Resistance) signaling in plants (Ekincialp et al. 2016).

Whey and AMF applications strongly modulated the antioxidant defense system of plants in response to ZYMV stress (Table 2). As expected, ZYMV infection induced oxidative stress, thereby triggering the plant’s natural defense responses (Radwan et al. 2006; Xu et al. 2024). However, combined applications such as WrMZ and WfMZ moderated this response to a more balanced level. These combinations particularly resulted in the highest levels of total phenolic content and proline.

This indicates the integration of two distinct defense mechanisms:

I. AMF colonization promoted the accumulation of non-enzymatic antioxidant pathways (phenolic compounds) and osmoprotective substances (proline), consistent with the literature suggesting that AMF enhances plant stress tolerance primarily through these pathways (Chun et al. 2018; Afshari et al. 2022; Chandrasekaran 2022; Boyno 2025).
II. Whey, through its bioactive compounds, activated phenylpropanoid pathways and ROS-scavenging enzymes, thereby strengthening the plant’s metabolic defense (Wang et al. 2012; Malos et al. 2025).

Overall, the synergy between Whey and AMF is thought to provide the plant with both enzymatic tools (CAT) to alleviate oxidative damage and precursors of structural defense compounds (phenolics, lignin via PAL) and osmoprotective compounds (proline) to maintain cellular turgor, thereby limiting viral spread.

It is well established that virus infection disrupts chloroplast structure, thereby reducing photosynthetic capacity (Zechmann et al. 2021), a phenomenon also confirmed in our study. Notably, the WfMZ combination (foliar whey + mycorrhiza) maintained high chlorophyll content despite ZYMV stress, demonstrating that this treatment mitigated virus-induced physiological impairments (Table 2). The nitrogenous compounds and microelements provided by foliar whey directly supported chlorophyll biosynthesis and stabilized the plant’s energy metabolism (Ekincialp et al. 2016), while AMF enhanced this metabolic balance through optimized nutrient and water uptake from the roots (Bhantana et al. 2021). This physiological buffering established a critical foundation for the plant to sustain essential metabolic functions while combating the virus. Furthermore, soil analyses clearly indicated the positive impact of this integration on organic matter content, moisture levels, and particularly phosphorus mobilization (Table 2). Although AMF contribute to phosphorus acquisition primarily by extending the absorptive surface area of the root system and enhancing inorganic phosphate uptake, they have limited intrinsic capacity to mineralize organic phosphorus due to the absence of phytase-encoding genes (Tisserant et al. 2013). Therefore, the mobilization of organic P in the rhizosphere is largely dependent on interactions between AMF and phosphate-solubilizing bacteria, which function synergistically to enhance nutrient availability (Jiang et al. 2021; Wang et al. 2023). In this context, the carbon-rich input supplied by whey may have stimulated rhizosphere microbial communities, indirectly promoting organic P mineralization and facilitating AMF-mediated nutrient acquisition (Akay and Sert 2020). Whey not only supplied a carbon source to stimulate microbial activity and support AMF symbiosis but also may have contributed to improved soil moisture retention. Rather than directly attributing the observed effects to specific biochemical buffering mechanisms, our results indicate that the combined application of whey and AMF was associated with a more stable rhizosphere environment under ZYMV stress. The observed decline in soil pH during infection—possibly linked to proton release and organic acid accumulation (Borhannuddin Bhuyan et al. 2019; Panchal et al. 2021)—may have imposed additional stress on plants. The improved plant performance observed in treated groups suggests that AMF–whey interactions could help mitigate such stress conditions; however, the precise mechanisms underlying these effects remain unclear. Further studies are needed to elucidate the specific biochemical and microbial pathways involved in whey-mediated modulation of rhizosphere processes.

The most notable finding of our study is that foliar application of whey (Wf) significantly reduced disease severity (Fig. S2). This demonstrates that whey functions not merely as a topical nutrient supplement but also triggers a systemic defense response in the plant, establishing a “primed state.” Although whey contains bioactive components such as peptides, amino acids, and lactate (Madureira et al. 2010; Macwan et al. 2016), direct evidence demonstrating activation of the salicylic acid (SA) pathway in planta following whey application is currently limited. Therefore, rather than attributing the observed effects to a specific SA-mediated mechanism, our results suggest that foliar whey application may enhance plant defense responses via yet-to-be-elucidated signaling pathways. SA-mediated systemic acquired resistance (SAR) is typically effective against biotrophic pathogens and viruses and can restrict viral movement by reinforcing cell walls (Baebler et al. 2014; Wang et al. 2022). However, further molecular analyses would be required to determine whether whey directly modulates SA-dependent signaling in this system. AMF are known to induce jasmonic acid (JA) and ethylene (ET)-based induced systemic resistance (ISR) in roots (Yu et al. 2022; Boyno et al. 2025a; Lidoy et al. 2025). Thus, while the combined WfMZ treatment showed enhanced protection, the potential integration of multiple defense signaling pathways (e.g., SA and JA/ET crosstalk) should be considered a working hypothesis rather than a demonstrated mechanism in the present study.

When evaluated together, the multivariate analyses (Correlation, PCA, and Heatmap) robustly confirmed the study’s core dynamics (Figs. 3, and 4; Fig. S3). All three analyses revealed a clear “growth-defense trade-off” between plant development parameters (biomass, chlorophyll) and biochemical defense responses (proline, phenolics, PAL). ZYMV infection suppressed plant growth while simultaneously activating defense mechanisms (Sipahioğlu et al. 2017; Hao et al. 2024). Within this overall framework, mycorrhizal applications were distinguished along a positive axis associated with plant phosphorus (P) uptake. The most notable finding is that, even under ZYMV stress, treatments combining whey and AMF (WfMZ, WrMZ) elicited the strongest and most coordinated biochemical defense responses. This demonstrates that these synergistic applications maximize the plant’s defense potential, thereby enhancing resilience against stress (Erman et al. 2011; Demir et al. 2015; Boyno et al. 2025b; Malos et al. 2025).

## Conclusion

This study demonstrates that the combined application of whey and arbuscular mycorrhizal fungi (AMF) can alleviate the negative effects of Zucchini yellow mosaic virus (ZYMV) on squash by improving plant growth, physiological performance, and antioxidant responses. The foliar whey + mycorrhiza (WfMZ) treatment showed the most consistent protective effects under viral stress. Rather than acting through a single mechanism, the observed benefits likely result from complementary effects on plant nutrition, physiological stability, and enhanced stress-associated biochemical responses. However, this study was conducted in a controlled climate chamber using a single host plant species (*Cucurbita pepo* L.) and a single AMF species (*Funneliformis mosseae*), which may limit the generalizability of the findings. In addition, the underlying molecular and rhizosphere-level mechanisms were not directly investigated. Future research should explore the applicability of this integrated approach in other plant species and against different pathogens, as well as investigate the underlying signaling and microbial processes involved. Such studies will be essential for better defining the role of whey–AMF combinations in sustainable, biologically based plant protection strategies.

## Electronic Supplementary Material

Below is the link to the electronic supplementary material.

Supplementary Material 1

Supplementary Material 2

Supplementary Material 3

Supplementary Material 4

Supplementary Material 5

Supplementary Material 6

## Data Availability

The data that support the findings of this study are available in Tables S1–S5 (Word format) and Figure S1–S3. The PCA data in this manuscript is available in Excel format (Notes S1).
