# Supplementary figures and images for: Integration of whey and mycorrhizal symbiosis: a sustainable biocontrol strategy against Zucchini yellow mosaic virus in squash
Source: Mycorrhiza. 2026 Apr 24;36(3):18. doi: 10.1007/s00572-026-01262-7 (PMC13109265; doi:10.1007/s00572-026-01262-7)

## Molecular Studies and Images

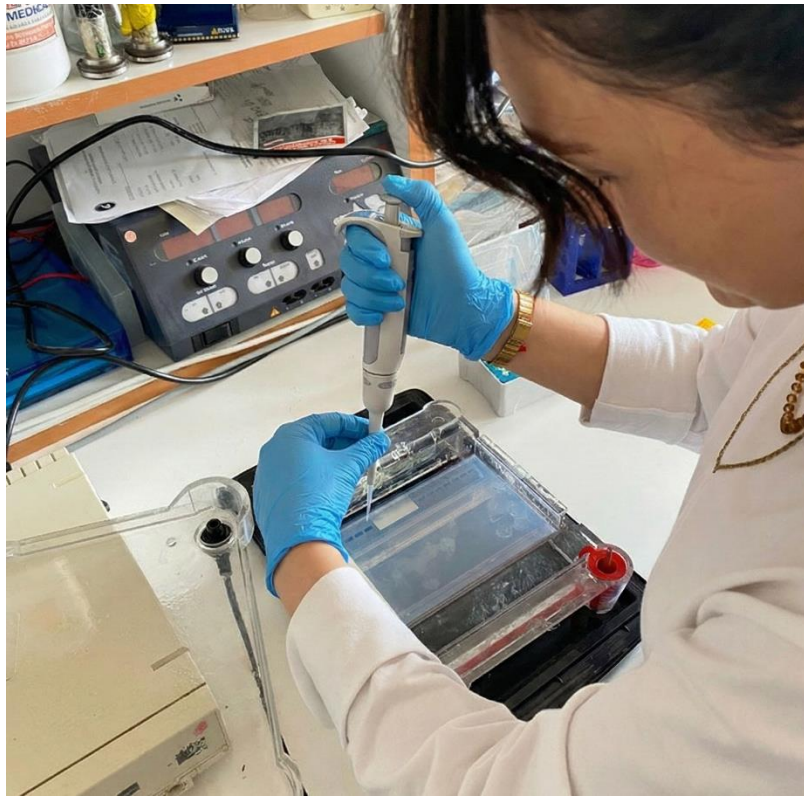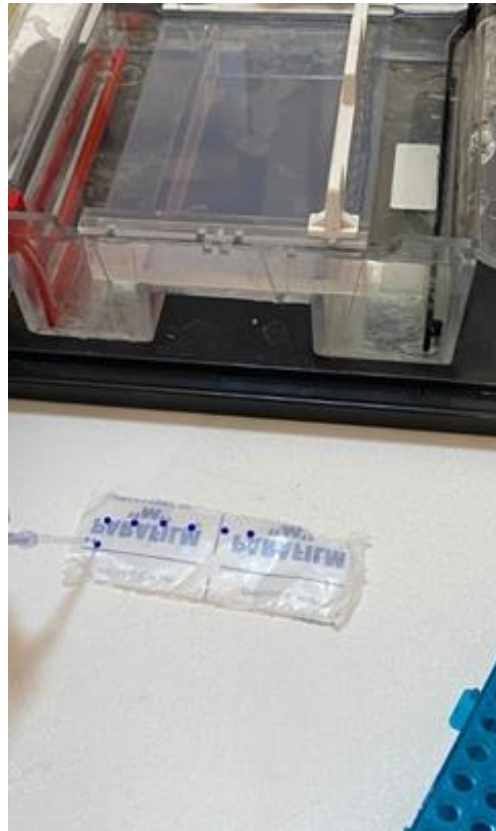

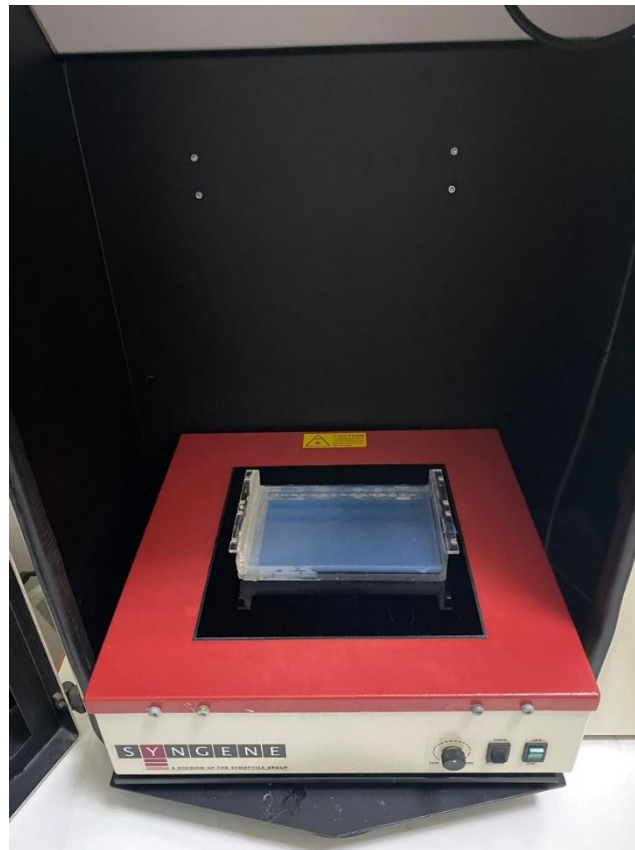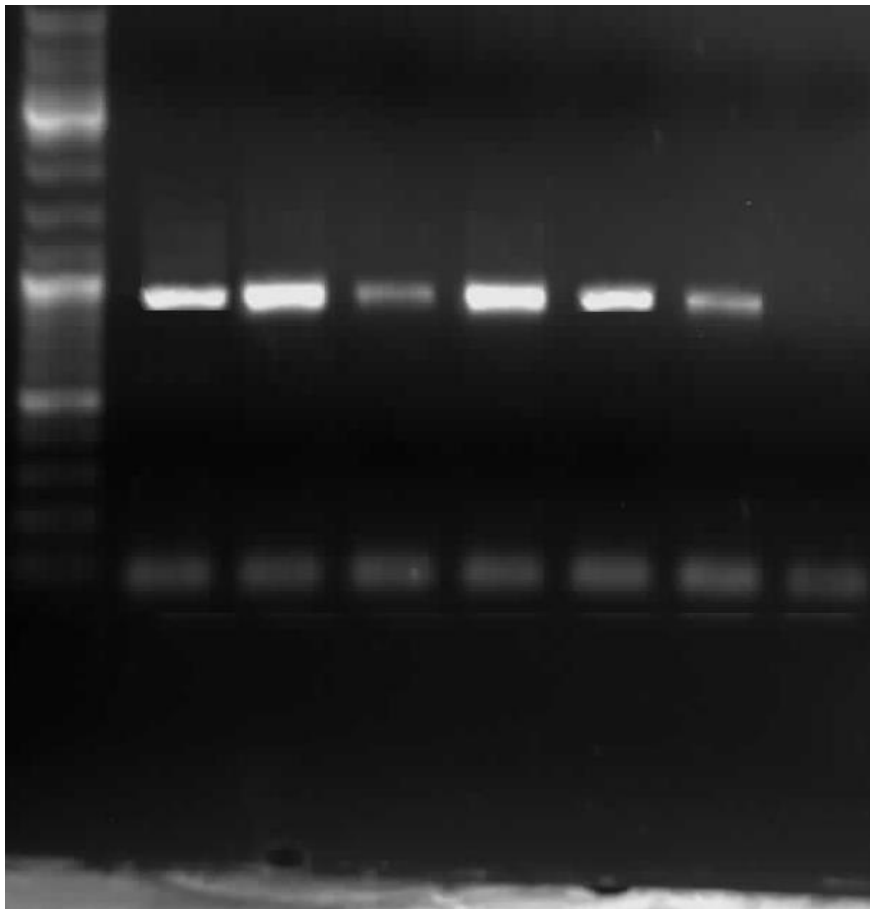

Some visual evidence of ZYMV

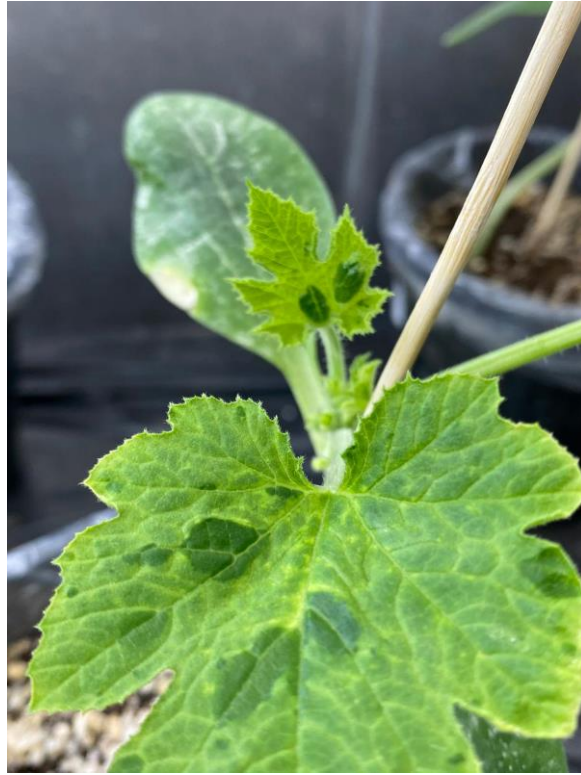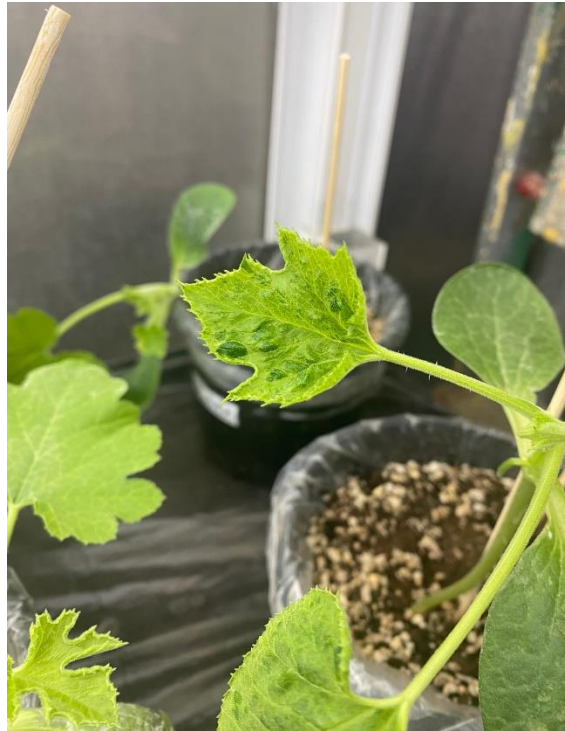

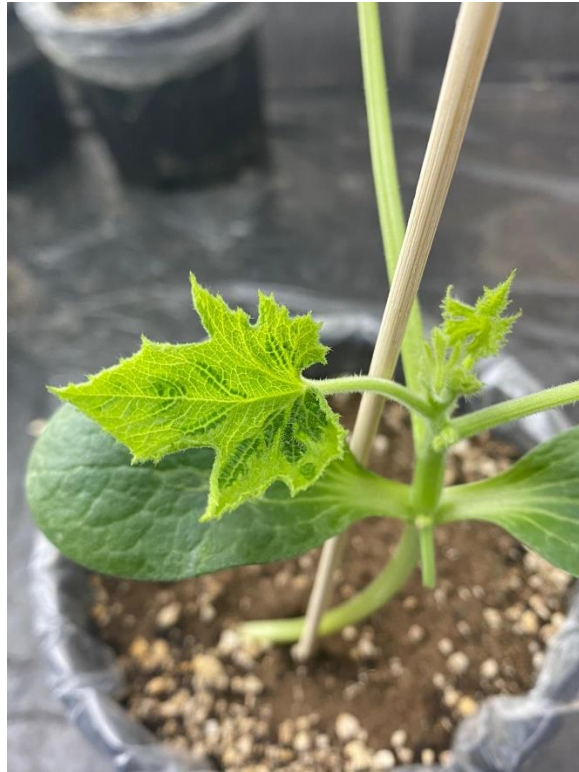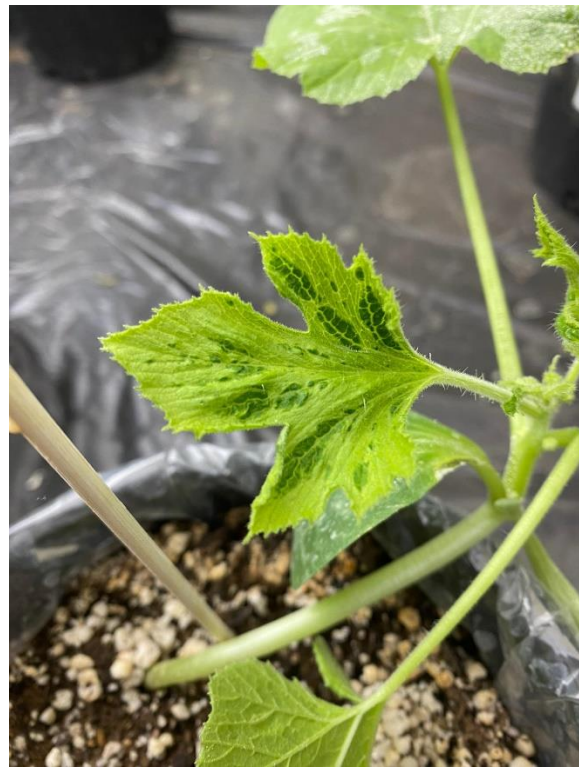

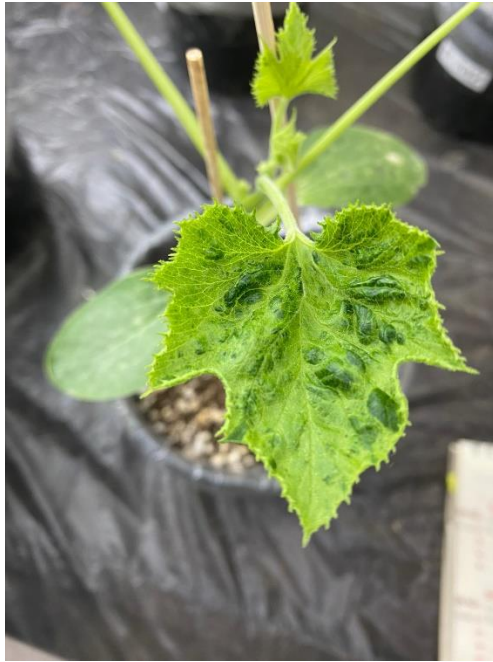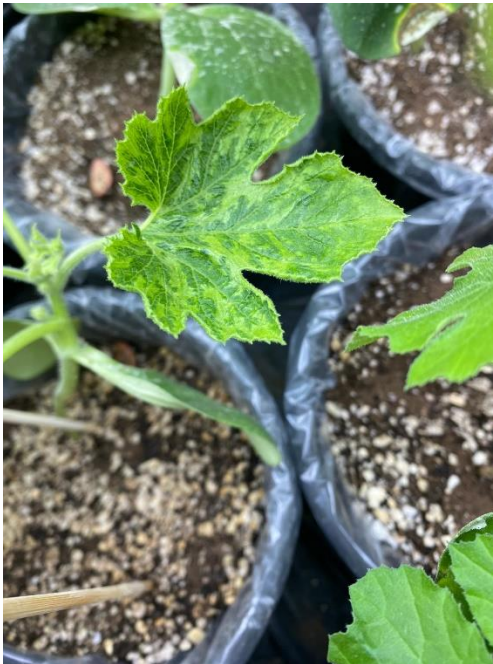

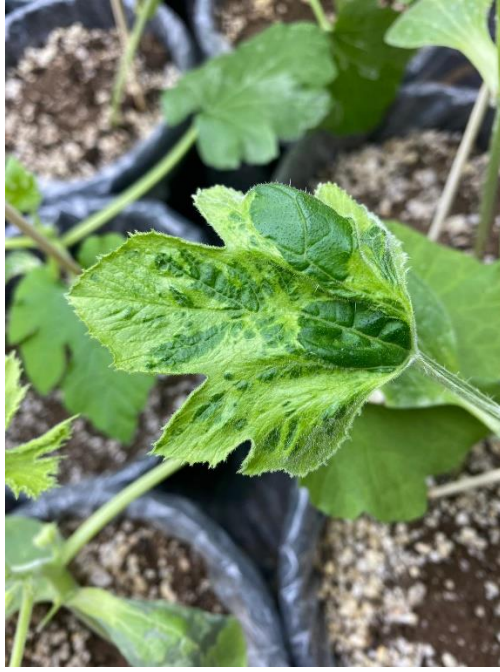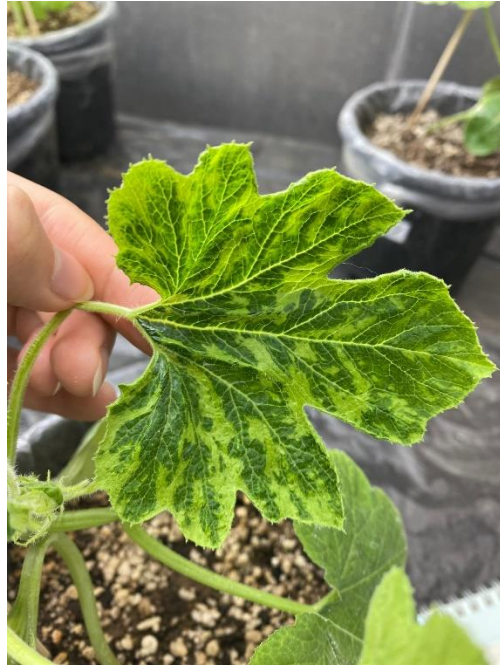

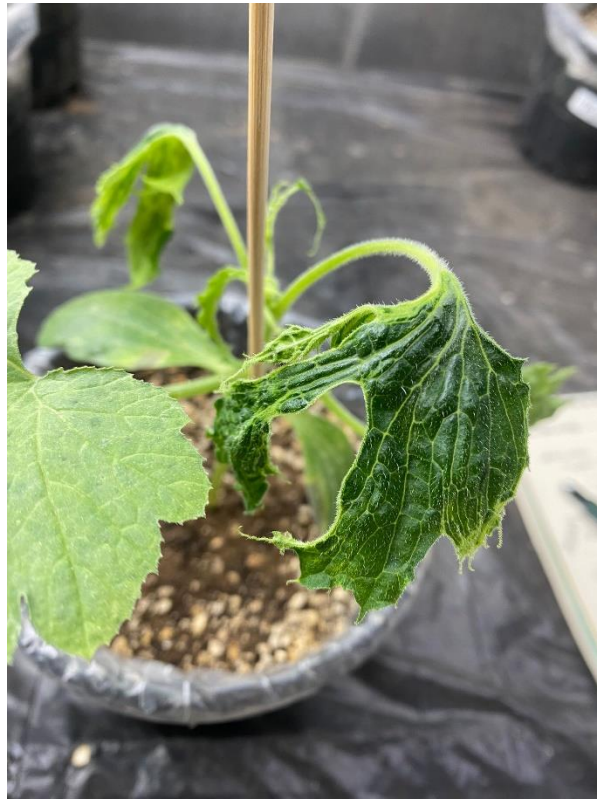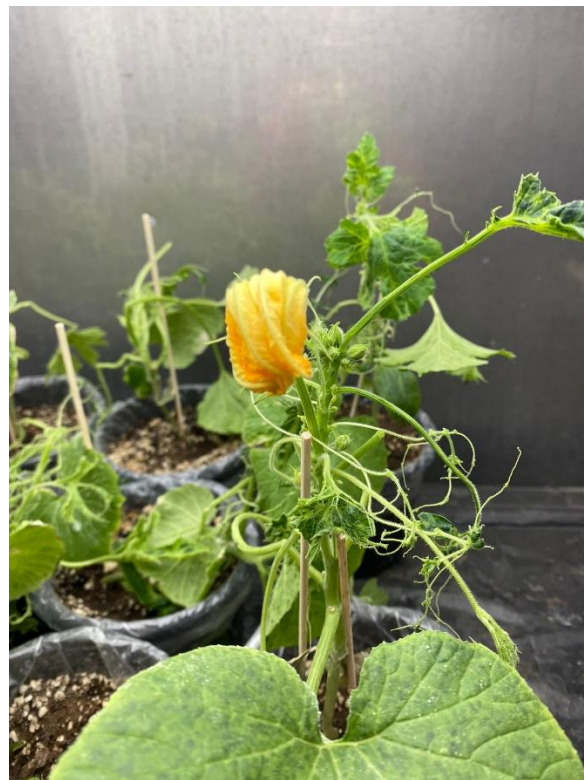

Supplement: Supplementary file 2 — Supplementary Material 2 [file 572_2026_1262_MOESM2_ESM.pdf]

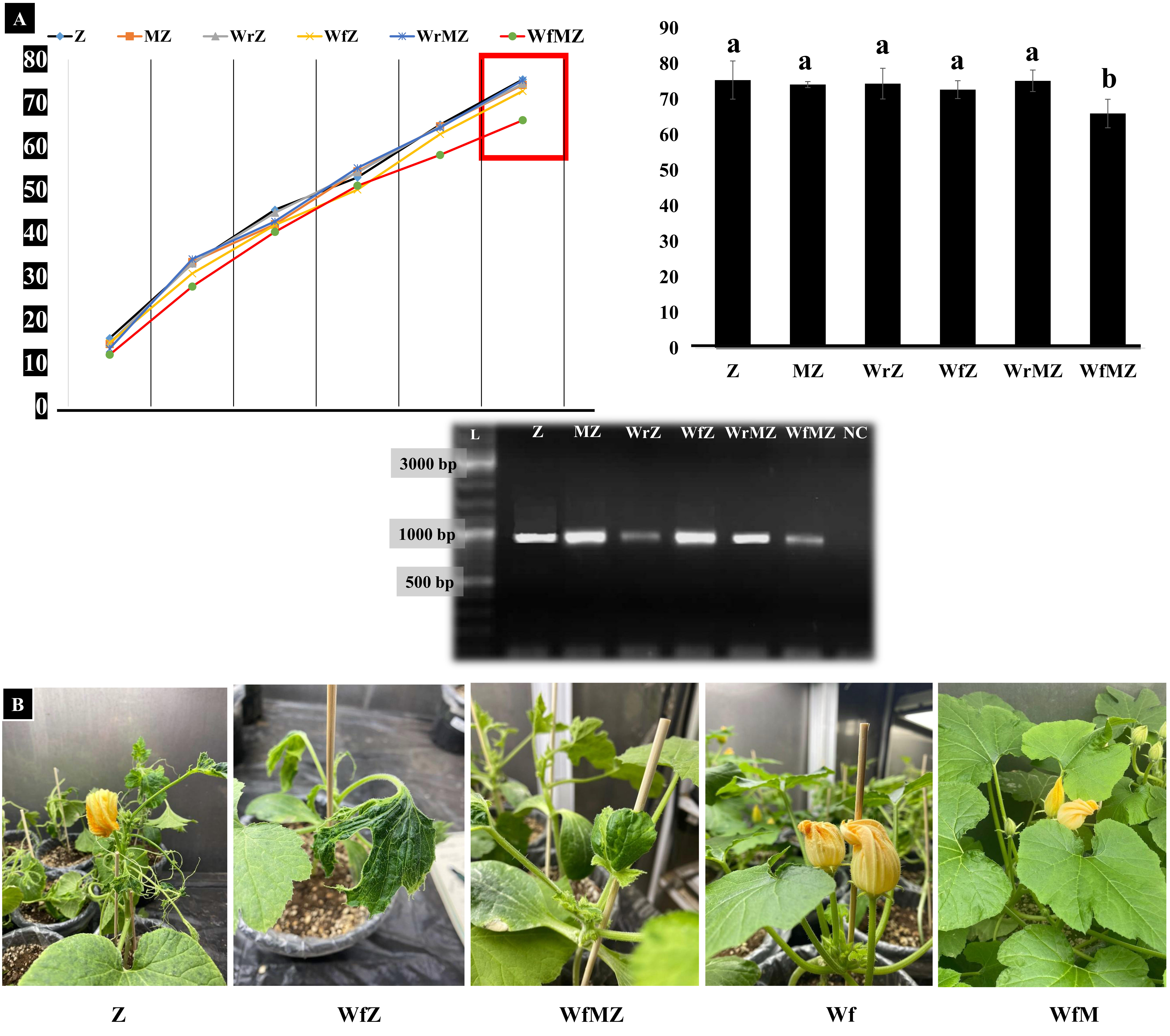

Supplement: Supplementary file 4 — Supplementary Material 4 [file 572_2026_1262_MOESM4_ESM.jpg]

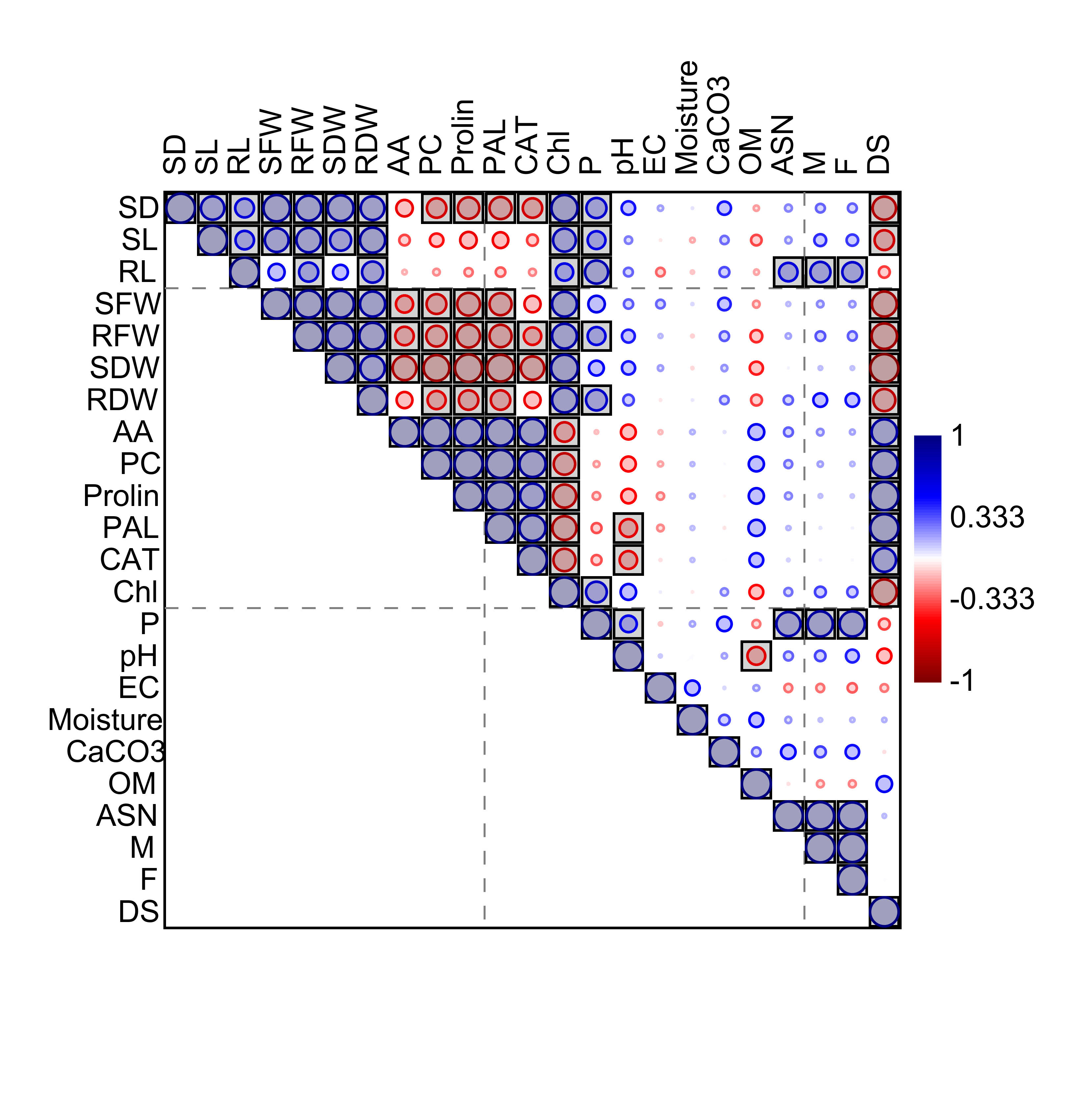

Supplement: Supplementary file 5 — Supplementary Material 5 [file 572_2026_1262_MOESM5_ESM.jpg]

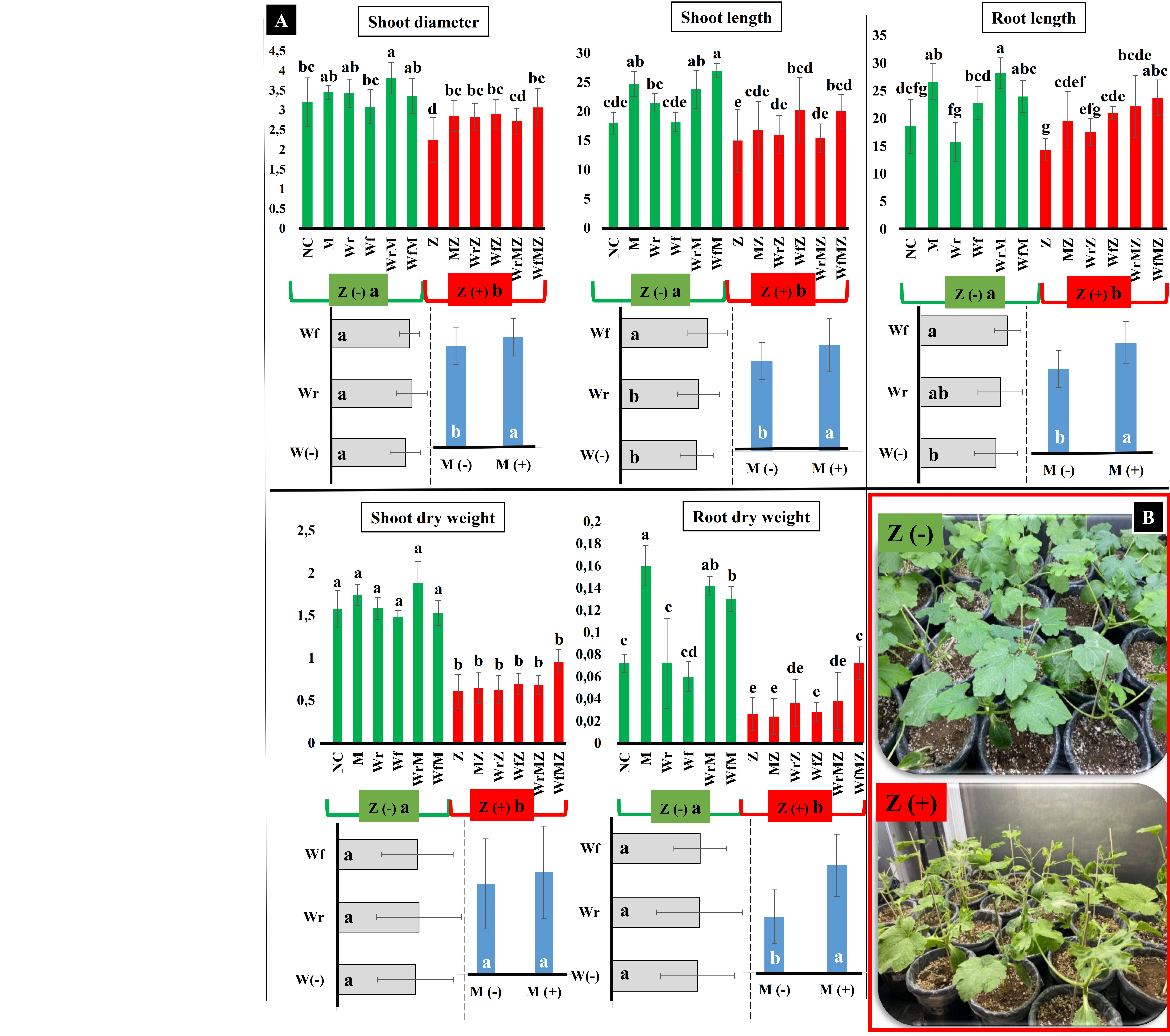

Supplement: Supplementary file 6 — Supplementary Material 6 [file 572_2026_1262_MOESM6_ESM.jpg]
