# Supplementary material for: Integration of whey and mycorrhizal symbiosis: a sustainable biocontrol strategy against Zucchini yellow mosaic virus in squash
Source: Mycorrhiza. 2026 Apr 24;36(3):18. doi: 10.1007/s00572-026-01262-7 (PMC13109265; doi:10.1007/s00572-026-01262-7)
Supplement: Supplementary file 3 — Supplementary Material 3 [file 572_2026_1262_MOESM3_ESM.docx]

**Table S1.** Treatment Codes and Descriptions

| **Code** | **Treatment Description** |
| --- | --- |
| **Z** | ZYMV only |
| **WrZ** | Rhizosphere-applied whey + ZYMV |
| **WfZ** | Foliar-applied whey + ZYMV |
| **MZ** | AMF + ZYMV |
| **WrMZ** | Rhizosphere-applied whey + AMF + ZYMV |
| **WfMZ** | Foliar-applied whey + AMF + ZYMV |
| **NC** | Negative Control (Healthy) |
| **Wr** | Rhizosphere-applied whey |
| **Wf** | Foliar-applied whey |
| **M** | AMF |
| **WrM** | Rhizosphere-applied whey + AMF |
| **WfM** | Foliar-applied whey + AMF |

**Table S2.** List of morphological, physiological, biochemical, soil, and mycorrhizal parameters measured in zucchini plants under different whey and AMF treatments. The table summarizes each parameter, its abbreviation, and the corresponding measurement unit used throughout the study.

| **Abbreviation** | **Parameter** | **Unit** |
| --- | --- | --- |
| **SD** | Stem Diameter | mm |
| **SL** | Shoot Length | cm |
| **RL** | Root Length | cm |
| **SFW** | Shoot Fresh Weight | g |
| **RFW** | Root Fresh Weight | g |
| **SDW** | Shoot Dry Weight | g |
| **RDW** | Root Dry Weight | g |
| **AA** | Total Antioxidant Activity | µmol Trolox g⁻¹ FW |
| **PC** | Total Phenolic Content | mg GAE g⁻¹ FW |
| **Proline** | Proline Content | µmol g⁻¹ FW |
| **PAL** | Phenylalanine Ammonia-Lyase | U mg⁻¹ protein |
| **CAT** | Catalase | µmol H₂O₂ min⁻¹ mg⁻¹ protein |
| **Chl** | Total Chlorophyll | SPAD |
| **P** | Plant Phosphorus Content | mg kg⁻¹ DW |
| **pH** | Soil pH | – |
| **EC** | Soil Electrical Conductivity | dS m⁻¹ |
| **Moisture** | Soil Moisture | % |
| **CaCO₃** | Calcium Carbonate | % |
| **OM** | Organic Matter | % |
| **ASN** | AMF Spore Number | spores g⁻¹ soil |
| **M** | Mycorrhizal Intensity | % |
| **F** | Mycorrhizal Frequency | % |
| **DS** | Disease Severity | % |

**Table S3.** Detailed methodological procedures used in the experiment

| **Assessment of plant biometric parameters**  At the end of the experimental period, plant growth parameters were thoroughly evaluated. In this context, the fresh and dry weights (g) of shoots and roots, shoot and root lengths (cm), and stem diameter (mm) were determined. For the measurement of fresh weights, the aerial parts of the seedlings were excised and weighed, while roots were carefully washed with tap water and reweighed. The obtained plant materials were then oven-dried at 70 °C for 48 h to determine their dry weights. Stem diameter was measured using a digital caliper (Insize-1112–150, Germany). |
| --- |
| **Assessment of Mycorrhizal Parameters**  At the end of the experiment, mycorrhizal colonization in Squash roots was determined using a modified version of the method described by Phillips and Hayman (1970), as adapted by Boyno (2024). Root samples were first cleared with 10% KOH (Merck 1.05012.1000, Germany) at 70 °C for 20 minutes and subsequently acidified with 10% HCl (Merck 1.00312.2500, Germany). The roots were then stained with 0.05% lactophenol blue solution (Merck 1.13741.0100, Germany). After staining, the roots were preserved in lactoglycerol solution, and microscope slides were prepared for observation.  **Mycorrhizal frequency (%F)** was calculated as the ratio of colonized root fragments to the total number of observed fragments. **Mycorrhizal intensity (%M)** was assessed using the 0–5 scale proposed by Trouvelot et al. (1986), where: 0 = no propagules; 1 = <10%; 2 = 10–30%; 3 = 30–50%; 4 = 50–90%; and 5 = 90–100% presence of propagules. The mycorrhizal intensity was calculated according to the following equation:  %M=[(95×n5)+(70×n4)+(30×n3)+(5×n2)+(1×n1)]÷N  Where:  *n₁–n₅* = represent the number of roots in each class  *N* = the total number of observed root segments.  The AMF spore density in the rhizosphere was determined using the ultrasonic centrifugation method described by Boyno et al. (2023). One gram of rhizosphere soil was suspended in 20 mL of distilled water and stirred for 5 minutes. The suspension was subjected to ultrasonic treatment at 28 kHz for 30 seconds (UltraClean12 model, Hydraultrasonic Co. Istanbul, Türkiye). After centrifugation, the supernatant was filtered into Petri dishes, and spores were counted under a stereomicroscope. The total number of AMF spores per gram of soil was calculated according to the following formula (Boyno et al. 2023):  TSN=[SN×W]÷S  where:  TSN = total number of AMF spores per gram of rhizosphere soil  SN = number of AMF spores in 1 mL of suspension  W = total suspension volume (mL)  S = weight of soil used (g) |
| **Assessment of Plant Biochemical Activity**  At the end of the experiment, 5 g of fresh leaf samples were homogenized with methanol and incubated in the dark at +4 °C for 14–16 h. The liquid phase was then separated by centrifugation, and the resulting supernatant was used for biochemical analyses.  The **total phenolic content (TPC)** was determined using the Folin–Ciocalteu colorimetric method described by Swain and Hillis (1959). For this purpose, 150 μL of Folin–Ciocalteu reagent and 300 μL of 20% sodium carbonate (Na₂CO₃) solution were added to the extract, followed by incubation in the dark for 30 min. Absorbance was measured at 725 nm using a UV–Vis spectrophotometer, and the total phenolic content was calculated accordingly.  The **total antioxidant activity (TAA)** was analyzed using the ferric reducing antioxidant power (FRAP) assay, following the method of Benzie and Strain (1996). A mixture of 150 μL of extract and 2850 μL of FRAP reagent was incubated at 25 °C for 30 min, and absorbance was recorded at 593 nm.  The **proline content** was determined according to the method of Bates et al. (1973) using the ninhydrin reagent. Briefly, 2 mL of the extract was mixed with 2 mL of 3% orthophosphoric acid, 2 mL of ninhydrin reagent, and 2 mL of glacial acetic acid in glass tubes. The reaction mixture was incubated in a boiling water bath (100 °C) for 1 h, rapidly cooled in an ice bath, and then 4 mL of toluene was added. The chromophore-containing upper phase was separated and the absorbance was measured at 520 nm using a spectrophotometer. |
| **Assessment of Antioxidant and Defense-Related Enzyme Activities**  **Catalase (CAT) activity:** Catalase activity was determined according to the method described by Jebara et al. (2010) by monitoring the decrease in absorbance of H₂O₂ at 240 nm. The reaction mixture consisted of 50 mM phosphate buffer (pH 7.0) and 10 mM H₂O₂. The reaction was initiated by adding 20 μL of enzyme extract and incubating the mixture at 25 °C for 2 min. The reaction was terminated by adding 0.5 mL of 1 M HCl solution. CAT activity was calculated based on the rate of H₂O₂ decomposition using an extinction coefficient of 0.0392 mM⁻¹ cm⁻¹ and expressed as mmol of H₂O₂ decomposed per minute per mg of protein.  **Phenylalanine ammonia-lyase (PAL) activity:** Phenylalanine ammonia-lyase activity was assayed with minor modifications following the procedure of Bhattacharyya and Ward (1988). One gram of fresh plant tissue was homogenized in 3 mL of ice-cold 0.1 M trisodium borate buffer (pH 8.5). The homogenate was filtered through muslin cloth and centrifuged at 16,000 rpm for 15 min at 4 °C. The resulting supernatant was used as the enzyme source. PAL activity was determined spectrophotometrically by measuring the formation of trans-cinnamic acid from L-phenylalanine at 270 nm. The reaction mixture contained 0.1 mL enzyme extract, 0.5 mL of 0.1 M trisodium borate buffer (pH 8.5), and 0.5 mL of 12 mM L-phenylalanine, adjusted to a final volume of 3 mL with deionized water. The mixture was incubated at 37 °C for 30 min, and the increase in absorbance at 270 nm was recorded for 5 min. All assays were performed in triplicate, and enzyme activity was expressed as ΔA₂₇₀ nm min⁻¹ mg⁻¹ protein. |
| **Assessment of Total Phosphorus and Chlorophyll Content in Plants**  The total phosphorus content in the plant tissues was determined using the vanadomolybophosphoric yellow method described by Barton (1948). For this purpose, 0.5 g of ground plant sample was placed into porcelain crucibles, and 1 mL of ethyl alcohol was added for pre-ashing. The samples were then ashed in a muffle furnace, after which 4 mL of hydrochloric acid (HCl) was added. Following heating at 80–90 °C for 15–20 minutes, the samples were filtered into volumetric flasks and brought to a final volume of 50 mL with distilled water. In the final step, toluene was added for phosphorus determination, and absorbance was measured spectrophotometrically at 430 nm.  The total chlorophyll content was determined using a SPAD meter (502-Plus, Konica Minolta, Japan). Measurements were taken from three fully expanded leaves per plant to represent the overall chlorophyll content of each plant (Fischer, 2001). |
| **Assessment of Soil Parameters**  **Soil organic matter (OM)** content was determined according to the method described by Nelson and Sommers (1982). In this method, organic matter was oxidized using potassium dichromate (K₂Cr₂O₇), and the excess dichromate was titrated with ferrous sulfate (FeSO₄) to calculate the organic matter content.  The **total lime content** was measured following the procedure described by Gülçur (1974), in which **calcium carbonate (CaCO₃)** in the soil reacts with hydrochloric acid (HCl), and the amount of released carbon dioxide (CO₂) is quantified.  **Soil moisture** content was determined using the gravimetric method described by Schmugge et al. (1980). A known amount of soil sample was oven-dried at 105 °C until a constant weight was achieved, and the moisture content was calculated based on the weight loss.  **Soil pH and electrical conductivity (EC)** were determined according to the method of Jackson (1958). For these measurements, a soil-to-water suspension was prepared at a ratio of 1:2.5 (w/v); pH was measured using a pH meter, and EC was determined using a conductivity meter. |
| **Disease Severity and Molecular Confirmation**  Following whey treatments, disease severity was assessed weekly (six times in total). Disease severity (DS) caused by *Zucchini yellow mosaic virus* (ZYMV) was rated using a 0–5 scale: 0 = no symptoms; 1 = <50% leaf mottling; 2 = >50% leaf mottling; 3 = mottling and mosaic; 4 = mosaic and leaf deformation; 5 = severe symptoms including filiform leaves (Xu et al. 2004). The percentage of disease severity was calculated using the Townsend Heubergeb formula (Townsend and Heubergeb 1943):  % DS=[(Scale value×Number of leaves at that scale)÷Total number of leaves]×100  Leaf samples from each treatment group were pooled and homogenized in a chilled mortar prior to total nucleic acid extraction. Total nucleic acids were isolated from 100 mg of squash leaf tissue using the silica capture method described by Foissac et al. (2001). Molecular diagnosis of ZYMV was performed |

**Table S4. RT-PCR Conditions and Primer Information for ZYMV Detection**

| **Parameter** | **Description** |
| --- | --- |
| **RT-PCR Method** | Two-step RT-PCR (Commercial Kit, Fermentas, Lithuania) |
| **Target Gene** | Coat Protein (CP) gene of ZYMV |
| **Primer Design Software** | Primer3 (<http://frodo.wi.mit.edu/primer3/>) |
| **Forward Primer (Z-F)** | 5’-TCAGGCACTCAGCCAACT-3’ |
| **Reverse Primer (Z-R)** | 5’-CTGCATTGTATTCACACCTAGT-3’ |

**Table S5** Between-Subjects Effects of Whey Application, AMF Inoculation, and ZYMV Infection on All Measured Plant and Soil Parameters

| **Tests of Between-Subjects Effects** | | | | | |
| --- | --- | --- | --- | --- | --- |
| Dependent Variable: Stem Diameter | | | | | |
| Source | Type III Sum of Squares | df | Mean Square | F | Sig. |
| Corrected Model | 9,292^a^ | 11 | ,845 | 4,729 | ,000 |
| Intercept | 568,691 | 1 | 568,691 | 3183,419 | ,000 |
| Whey App. | ,712 | 2 | ,356 | 1,994 | ,147 |
| M | 1,019 | 1 | 1,019 | 5,705 | ,021 |
| ZYMV | 5,791 | 1 | 5,791 | 32,416 | ,000 |
| Whey App. * M | ,219 | 2 | ,109 | ,612 | ,547 |
| Whey App. * ZYMV | 1,077 | 2 | ,538 | 3,014 | ,058 |
| M * ZYMV | ,028 | 1 | ,028 | ,158 | ,693 |
| Whey App. * M * ZYMV | ,446 | 2 | ,223 | 1,248 | ,296 |
| Error | 8,575 | 48 | ,179 |  |  |
| Total | 586,558 | 60 |  |  |  |
| Corrected Total | 17,867 | 59 |  |  |  |
| a. R Squared = ,520 (Adjusted R Squared = ,410) | | | | | |

| **Tests of Between-Subjects Effects** | | | | | |
| --- | --- | --- | --- | --- | --- |
| Dependent Variable: Shoot Length | | | | | |
| Source | Type III Sum of Squares | df | Mean Square | F | Sig. |
| Corrected Model | 832,636^a^ | 11 | 75,694 | 6,713 | ,000 |
| Intercept | 23334,676 | 1 | 23334,676 | 2069,403 | ,000 |
| Whey App. | 83,877 | 2 | 41,939 | 3,719 | ,031 |
| M | 148,051 | 1 | 148,051 | 13,130 | ,001 |
| ZYMV | 368,776 | 1 | 368,776 | 32,704 | ,000 |
| Whey App. * M | 39,402 | 2 | 19,701 | 1,747 | ,185 |
| Whey App. * ZYMV | 51,877 | 2 | 25,939 | 2,300 | ,111 |
| M * ZYMV | 116,901 | 1 | 116,901 | 10,367 | ,002 |
| Whey App. * M * ZYMV | 23,752 | 2 | 11,876 | 1,053 | ,357 |
| Error | 541,250 | 48 | 11,276 |  |  |
| Total | 24708,563 | 60 |  |  |  |
| Corrected Total | 1373,886 | 59 |  |  |  |
| a. R Squared = ,606 (Adjusted R Squared = ,516) | | | | | |

| **Tests of Between-Subjects Effects** | | | | | |
| --- | --- | --- | --- | --- | --- |
| Dependent Variable: Root Length | | | | | |
| Source | Type III Sum of Squares | df | Mean Square | F | Sig. |
| Corrected Model | 974,336^a^ | 11 | 88,576 | 6,964 | ,000 |
| Intercept | 27019,426 | 1 | 27019,426 | 2124,204 | ,000 |
| Whey App. | 95,990 | 2 | 47,995 | 3,773 | ,030 |
| M | 488,776 | 1 | 488,776 | 38,426 | ,000 |
| ZYMV | 128,334 | 1 | 128,334 | 10,089 | ,003 |
| Whey App. * M | 113,090 | 2 | 56,545 | 4,445 | ,017 |
| Whey App. * ZYMV | 58,581 | 2 | 29,291 | 2,303 | ,111 |
| M * ZYMV | 34,884 | 1 | 34,884 | 2,743 | ,104 |
| Whey App. * M * ZYMV | 54,681 | 2 | 27,341 | 2,149 | ,128 |
| Error | 610,550 | 48 | 12,720 |  |  |
| Total | 28604,313 | 60 |  |  |  |
| Corrected Total | 1584,886 | 59 |  |  |  |
| a. R Squared = ,615 (Adjusted R Squared = ,526) | | | | | |

| **Tests of Between-Subjects Effects** | | | | | |
| --- | --- | --- | --- | --- | --- |
| Dependent Variable: Shoot Fresh Weight | | | | | |
| Source | Type III Sum of Squares | df | Mean Square | F | Sig. |
| Corrected Model | 1207,018^a^ | 11 | 109,729 | 7,881 | ,000 |
| Intercept | 15066,377 | 1 | 15066,377 | 1082,049 | ,000 |
| Whey App. | 79,661 | 2 | 39,830 | 2,861 | ,067 |
| M | 69,811 | 1 | 69,811 | 5,014 | ,030 |
| ZYMV | 888,118 | 1 | 888,118 | 63,784 | ,000 |
| Whey App. * M | 2,482 | 2 | 1,241 | ,089 | ,915 |
| Whey App. * ZYMV | 98,102 | 2 | 49,051 | 3,523 | ,037 |
| M * ZYMV | 59,242 | 1 | 59,242 | 4,255 | ,045 |
| Whey App. * M * ZYMV | 9,601 | 2 | 4,800 | ,345 | ,710 |
| Error | 668,349 | 48 | 13,924 |  |  |
| Total | 16941,744 | 60 |  |  |  |
| Corrected Total | 1875,367 | 59 |  |  |  |
| a. R Squared = ,644 (Adjusted R Squared = ,562) | | | | | |

| **Tests of Between-Subjects Effects** | | | | | |
| --- | --- | --- | --- | --- | --- |
| Dependent Variable: Root Fresh Weight | | | | | |
| Source | Type III Sum of Squares | df | Mean Square | F | Sig. |
| Corrected Model | 3,631^a^ | 11 | ,330 | 18,267 | ,000 |
| Intercept | 10,517 | 1 | 10,517 | 581,982 | ,000 |
| Whey App. | ,047 | 2 | ,023 | 1,299 | ,282 |
| M | ,430 | 1 | ,430 | 23,801 | ,000 |
| ZYMV | 2,765 | 1 | 2,765 | 153,004 | ,000 |
| Whey App. * M | ,035 | 2 | ,017 | ,956 | ,392 |
| Whey App. * ZYMV | ,007 | 2 | ,004 | ,194 | ,824 |
| M * ZYMV | ,277 | 1 | ,277 | 15,353 | ,000 |
| Whey App. * M * ZYMV | ,070 | 2 | ,035 | 1,939 | ,155 |
| Error | ,867 | 48 | ,018 |  |  |
| Total | 15,015 | 60 |  |  |  |
| Corrected Total | 4,498 | 59 |  |  |  |
| a. R Squared = ,807 (Adjusted R Squared = ,763) | | | | | |

| **Tests of Between-Subjects Effects** | | | | | |
| --- | --- | --- | --- | --- | --- |
| Dependent Variable: Shoot Dry Weight | | | | | |
| Source | Type III Sum of Squares | df | Mean Square | F | Sig. |
| Corrected Model | 13,933^a^ | 11 | 1,267 | 15,035 | ,000 |
| Intercept | 81,853 | 1 | 81,853 | 971,573 | ,000 |
| Whey App. | ,024 | 2 | ,012 | ,143 | ,867 |
| M | ,308 | 1 | ,308 | 3,658 | ,062 |
| ZYMV | 12,974 | 1 | 12,974 | 153,991 | ,000 |
| Whey App. * M | ,014 | 2 | ,007 | ,085 | ,919 |
| Whey App. * ZYMV | ,466 | 2 | ,233 | 2,766 | ,073 |
| M * ZYMV | ,009 | 1 | ,009 | ,103 | ,750 |
| Whey App. * M * ZYMV | ,139 | 2 | ,069 | ,822 | ,446 |
| Error | 4,044 | 48 | ,084 |  |  |
| Total | 99,831 | 60 |  |  |  |
| Corrected Total | 17,977 | 59 |  |  |  |
| a. R Squared = ,775 (Adjusted R Squared = ,724) | | | | | |

| **Tests of Between-Subjects Effects** | | | | | |
| --- | --- | --- | --- | --- | --- |
| Dependent Variable: Root Dry Weight | | | | | |
| Source | Type III Sum of Squares | df | Mean Square | F | Sig. |
| Corrected Model | ,125^a^ | 11 | ,011 | 31,587 | ,000 |
| Intercept | ,308 | 1 | ,308 | 858,005 | ,000 |
| Whey App. | 4,333E-5 | 2 | 2,167E-5 | ,060 | ,942 |
| M | ,031 | 1 | ,031 | 85,828 | ,000 |
| ZYMV | ,071 | 1 | ,071 | 196,919 | ,000 |
| Whey App. * M | ,001 | 2 | ,001 | 1,592 | ,214 |
| Whey App. * ZYMV | ,005 | 2 | ,003 | 7,383 | ,002 |
| M * ZYMV | ,014 | 1 | ,014 | 39,276 | ,000 |
| Whey App. * M * ZYMV | ,003 | 2 | ,001 | 3,680 | ,033 |
| Error | ,017 | 48 | ,000 |  |  |
| Total | ,450 | 60 |  |  |  |
| Corrected Total | ,142 | 59 |  |  |  |
| a. R Squared = ,879 (Adjusted R Squared = ,851) | | | | | |

| **Tests of Between-Subjects Effects** | | | | | |
| --- | --- | --- | --- | --- | --- |
| Dependent Variable: Total Antioxidant Activity | | | | | |
| Source | Type III Sum of Squares | df | Mean Square | F | Sig. |
| Corrected Model | 28,991^a^ | 11 | 2,636 | 105,623 | ,000 |
| Intercept | 295,526 | 1 | 295,526 | 11843,560 | ,000 |
| Whey App. | 3,923 | 2 | 1,961 | 78,607 | ,000 |
| M | ,744 | 1 | ,744 | 29,805 | ,000 |
| ZYMV | 22,841 | 1 | 22,841 | 915,393 | ,000 |
| Whey App. * M | ,301 | 2 | ,151 | 6,038 | ,005 |
| Whey App. * ZYMV | ,479 | 2 | ,240 | 9,602 | ,000 |
| M * ZYMV | ,596 | 1 | ,596 | 23,886 | ,000 |
| Whey App. * M * ZYMV | ,107 | 2 | ,053 | 2,136 | ,129 |
| Error | 1,198 | 48 | ,025 |  |  |
| Total | 325,715 | 60 |  |  |  |
| Corrected Total | 30,189 | 59 |  |  |  |
| a. R Squared = ,960 (Adjusted R Squared = ,951) | | | | | |

| **Tests of Between-Subjects Effects** | | | | | |
| --- | --- | --- | --- | --- | --- |
| Dependent Variable: Total Phenolic Content | | | | | |
| Source | Type III Sum of Squares | df | Mean Square | F | Sig. |
| Corrected Model | 57,376^a^ | 11 | 5,216 | 108,468 | ,000 |
| Intercept | 465,206 | 1 | 465,206 | 9674,165 | ,000 |
| Whey App. | 3,595 | 2 | 1,798 | 37,384 | ,000 |
| M | ,945 | 1 | ,945 | 19,652 | ,000 |
| ZYMV | 50,362 | 1 | 50,362 | 1047,293 | ,000 |
| Whey App. * M | ,424 | 2 | ,212 | 4,414 | ,017 |
| Whey App. * ZYMV | 1,046 | 2 | ,523 | 10,880 | ,000 |
| M * ZYMV | ,737 | 1 | ,737 | 15,327 | ,000 |
| Whey App. * M * ZYMV | ,266 | 2 | ,133 | 2,762 | ,073 |
| Error | 2,308 | 48 | ,048 |  |  |
| Total | 524,890 | 60 |  |  |  |
| Corrected Total | 59,684 | 59 |  |  |  |
| a. R Squared = ,961 (Adjusted R Squared = ,952) | | | | | |

| **Tests of Between-Subjects Effects** | | | | | |
| --- | --- | --- | --- | --- | --- |
| Dependent Variable: Proline Content | | | | | |
| Source | Type III Sum of Squares | df | Mean Square | F | Sig. |
| Corrected Model | 2418,666^a^ | 11 | 219,879 | 1009,150 | ,000 |
| Intercept | 8369,511 | 1 | 8369,511 | 38412,515 | ,000 |
| Whey App. | 21,078 | 2 | 10,539 | 48,370 | ,000 |
| M | 20,721 | 1 | 20,721 | 95,101 | ,000 |
| ZYMV | 2347,752 | 1 | 2347,752 | 10775,187 | ,000 |
| Whey App. * M | 5,971 | 2 | 2,986 | 13,703 | ,000 |
| Whey App. * ZYMV | 5,578 | 2 | 2,789 | 12,800 | ,000 |
| M * ZYMV | 13,424 | 1 | 13,424 | 61,609 | ,000 |
| Whey App. * M * ZYMV | 4,142 | 2 | 2,071 | 9,505 | ,000 |
| Error | 10,458 | 48 | ,218 |  |  |
| Total | 10798,635 | 60 |  |  |  |
| Corrected Total | 2429,124 | 59 |  |  |  |
| a. R Squared = ,996 (Adjusted R Squared = ,995) | | | | | |

| **Tests of Between-Subjects Effects** | | | | | |
| --- | --- | --- | --- | --- | --- |
| Dependent Variable: Phenylalanine Ammonia-Lyase | | | | | |
| Source | Type III Sum of Squares | df | Mean Square | F | Sig. |
| Corrected Model | 1423,077^a^ | 11 | 129,371 | 251,331 | ,000 |
| Intercept | 5167,846 | 1 | 5167,846 | 10039,673 | ,000 |
| Whey App. | 15,415 | 2 | 7,708 | 14,974 | ,000 |
| M | ,123 | 1 | ,123 | ,240 | ,627 |
| ZYMV | 1327,281 | 1 | 1327,281 | 2578,533 | ,000 |
| Whey App. * M | 14,449 | 2 | 7,225 | 14,035 | ,000 |
| Whey App. * ZYMV | 5,716 | 2 | 2,858 | 5,553 | ,007 |
| M * ZYMV | 52,192 | 1 | 52,192 | 101,394 | ,000 |
| Whey App. * M * ZYMV | 7,900 | 2 | 3,950 | 7,674 | ,001 |
| Error | 24,708 | 48 | ,515 |  |  |
| Total | 6615,631 | 60 |  |  |  |
| Corrected Total | 1447,784 | 59 |  |  |  |
| a. R Squared = ,983 (Adjusted R Squared = ,979) | | | | | |

| **Tests of Between-Subjects Effects** | | | | | |
| --- | --- | --- | --- | --- | --- |
| Dependent Variable: Catalase | | | | | |
| Source | Type III Sum of Squares | df | Mean Square | F | Sig. |
| Corrected Model | 307,084^a^ | 11 | 27,917 | 680,812 | ,000 |
| Intercept | 6661,281 | 1 | 6661,281 | 162450,449 | ,000 |
| Whey App. | 19,499 | 2 | 9,750 | 237,768 | ,000 |
| M | 2,667E-5 | 1 | 2,667E-5 | ,001 | ,980 |
| ZYMV | 198,671 | 1 | 198,671 | 4845,048 | ,000 |
| Whey App. * M | 2,095 | 2 | 1,048 | 25,549 | ,000 |
| Whey App. * ZYMV | 43,896 | 2 | 21,948 | 535,248 | ,000 |
| M * ZYMV | 34,383 | 1 | 34,383 | 838,506 | ,000 |
| Whey App. * M * ZYMV | 8,539 | 2 | 4,270 | 104,124 | ,000 |
| Error | 1,968 | 48 | ,041 |  |  |
| Total | 6970,333 | 60 |  |  |  |
| Corrected Total | 309,052 | 59 |  |  |  |
| a. R Squared = ,994 (Adjusted R Squared = ,992) | | | | | |

| **Tests of Between-Subjects Effects** | | | | | |
| --- | --- | --- | --- | --- | --- |
| Dependent Variable: Plant Phosphorus Content | | | | | |
| Source | Type III Sum of Squares | df | Mean Square | F | Sig. |
| Corrected Model | 18,312^a^ | 11 | 1,665 | 38,489 | ,000 |
| Intercept | 586,250 | 1 | 586,250 | 13554,392 | ,000 |
| Whey App. | ,822 | 2 | ,411 | 9,498 | ,000 |
| M | 14,084 | 1 | 14,084 | 325,639 | ,000 |
| ZYMV | 2,231 | 1 | 2,231 | 51,584 | ,000 |
| Whey App. * M | ,151 | 2 | ,076 | 1,749 | ,185 |
| Whey App. * ZYMV | ,360 | 2 | ,180 | 4,164 | ,021 |
| M * ZYMV | ,157 | 1 | ,157 | 3,632 | ,063 |
| Whey App. * M * ZYMV | ,506 | 2 | ,253 | 5,850 | ,005 |
| Error | 2,076 | 48 | ,043 |  |  |
| Total | 606,638 | 60 |  |  |  |
| Corrected Total | 20,388 | 59 |  |  |  |
| a. R Squared = ,898 (Adjusted R Squared = ,875) | | | | | |

| **Tests of Between-Subjects Effects** | | | | | | | |
| --- | --- | --- | --- | --- | --- | --- | --- |
| **Dependent Variable: Total Chlorophyll** | | | | | | | |
| Source | Type III Sum of Squares | df | Mean Square | F | Sig. | |  |
| Corrected Model | 1503,072^a^ | 11 | 136,643 | 10,197 | ,000 | |  |
| Intercept | 71965,296 | 1 | 71965,296 | 5370,679 | ,000 | |  |
| Whey App. | 18,402 | 2 | 9,201 | ,687 | ,508 | |  |
| M | 219,804 | 1 | 219,804 | 16,404 | ,000 | |  |
| ZYMV | 1051,021 | 1 | 1051,021 | 78,436 | ,000 | |  |
| Whey App. * M | 49,325 | 2 | 24,663 | 1,841 | ,170 | |  |
| Whey App. * ZYMV | 122,458 | 2 | 61,229 | 4,569 | ,015 | |  |
| M * ZYMV | 19,176 | 1 | 19,176 | 1,431 | ,237 | |  |
| Whey App. * M * ZYMV | 22,886 | 2 | 11,443 | ,854 | ,432 | |  |
| Error | 643,184 | 48 | 13,400 |  |  | |  |
| Total | 74111,552 | 60 |  |  |  | |  |
| Corrected Total | 2146,256 | 59 |  |  |  | |  |
| a. R Squared = ,700 (Adjusted R Squared = ,632) | | | | | | |  |
| **Tests of Between-Subjects Effects** | | | | | | | |
| Dependent Variable: Soil pH | | | | | | | |
| Source | Type III Sum of Squares | df | Mean Square | F | | Sig. | |
| Corrected Model | ,291^a^ | 11 | ,026 | 2,596 | | ,025 | |
| Intercept | 2165,196 | 1 | 2165,196 | 212679,553 | | ,000 | |
| Whey App. | ,011 | 2 | ,006 | ,544 | | ,588 | |
| M | ,057 | 1 | ,057 | 5,580 | | ,027 | |
| ZYMV | ,074 | 1 | ,074 | 7,249 | | ,013 | |
| Whey App. * M | ,018 | 2 | ,009 | ,892 | | ,423 | |
| Whey App. * ZYMV | ,038 | 2 | ,019 | 1,863 | | ,177 | |
| M * ZYMV | ,058 | 1 | ,058 | 5,737 | | ,025 | |
| Whey App. * M * ZYMV | ,035 | 2 | ,017 | 1,698 | | ,204 | |
| Error | ,244 | 24 | ,010 |  | |  | |
| Total | 2165,731 | 36 |  |  | |  | |
| Corrected Total | ,535 | 35 |  |  | |  | |
| a. R Squared = ,543 (Adjusted R Squared = ,334) | | | | | | | |
| **Tests of Between-Subjects Effects** | | | | | | |  |
| Dependent Variable: Soil Electrical Conductivity | | | | | | |  |
| Source | Type III Sum of Squares | df | Mean Square | F | Sig. | |  |
| Corrected Model | 9225,889^a^ | 11 | 838,717 | 3,081 | ,010 | |  |
| Intercept | 890506,778 | 1 | 890506,778 | 3271,249 | ,000 | |  |
| Whey App. | 5713,556 | 2 | 2856,778 | 10,494 | ,001 | |  |
| M | 981,778 | 1 | 981,778 | 3,607 | ,070 | |  |
| ZYMV | 821,778 | 1 | 821,778 | 3,019 | ,095 | |  |
| Whey App. * M | 64,889 | 2 | 32,444 | ,119 | ,888 | |  |
| Whey App. * ZYMV | 1243,556 | 2 | 621,778 | 2,284 | ,124 | |  |
| M * ZYMV | 40,111 | 1 | 40,111 | ,147 | ,704 | |  |
| Whey App. * M * ZYMV | 360,222 | 2 | 180,111 | ,662 | ,525 | |  |
| Error | 6533,333 | 24 | 272,222 |  |  | |  |
| Total | 906266,000 | 36 |  |  |  | |  |
| Corrected Total | 15759,222 | 35 |  |  |  | |  |
| a. R Squared = ,585 (Adjusted R Squared = ,395) | | | | | | |  |

| **Tests of Between-Subjects Effects** | | | | | |
| --- | --- | --- | --- | --- | --- |
| Dependent Variable: Soil Moisture | | | | | |
| Source | Type III Sum of Squares | df | Mean Square | F | Sig. |
| Corrected Model | 2,489^a^ | 11 | ,226 | 7,874 | ,000 |
| Intercept | 96,629 | 1 | 96,629 | 3362,305 | ,000 |
| Whey App. | 2,043 | 2 | 1,021 | 35,537 | ,000 |
| M | ,056 | 1 | ,056 | 1,949 | ,175 |
| ZYMV | ,046 | 1 | ,046 | 1,584 | ,220 |
| Whey App. * M | ,157 | 2 | ,078 | 2,731 | ,085 |
| Whey App. * ZYMV | ,006 | 2 | ,003 | ,104 | ,902 |
| M * ZYMV | ,022 | 1 | ,022 | ,749 | ,396 |
| Whey App. * M * ZYMV | ,161 | 2 | ,080 | 2,795 | ,081 |
| Error | ,690 | 24 | ,029 |  |  |
| Total | 99,808 | 36 |  |  |  |
| Corrected Total | 3,179 | 35 |  |  |  |
| a. R Squared = ,783 (Adjusted R Squared = ,684) | | | | | |

| **Tests of Between-Subjects Effects** | | | | | |
| --- | --- | --- | --- | --- | --- |
| Dependent Variable: CACO3 Calcium Carbonate | | | | | |
| Source | Type III Sum of Squares | df | Mean Square | F | Sig. |
| Corrected Model | 82,412^a^ | 11 | 7,492 | 2,264 | ,046 |
| Intercept | 3138,987 | 1 | 3138,987 | 948,429 | ,000 |
| Whey App. | 12,843 | 2 | 6,422 | 1,940 | ,166 |
| M | 18,749 | 1 | 18,749 | 5,665 | ,026 |
| ZYMV | ,336 | 1 | ,336 | ,102 | ,753 |
| Whey App. * M | 1,821 | 2 | ,911 | ,275 | ,762 |
| Whey App. * ZYMV | 30,618 | 2 | 15,309 | 4,625 | ,020 |
| M * ZYMV | 2,382 | 1 | 2,382 | ,720 | ,405 |
| Whey App. * M * ZYMV | 15,663 | 2 | 7,832 | 2,366 | ,115 |
| Error | 79,432 | 24 | 3,310 |  |  |
| Total | 3300,832 | 36 |  |  |  |
| Corrected Total | 161,844 | 35 |  |  |  |
| a. R Squared = ,509 (Adjusted R Squared = ,284) | | | | | |

| **Tests of Between-Subjects Effects** | | | | | |
| --- | --- | --- | --- | --- | --- |
| Dependent Variable: Organic Matter | | | | | |
| Source | Type III Sum of Squares | df | Mean Square | F | Sig. |
| Corrected Model | 24,021^a^ | 11 | 2,184 | 3,892 | ,003 |
| Intercept | 523,189 | 1 | 523,189 | 932,481 | ,000 |
| Whey App. | 8,144 | 2 | 4,072 | 7,257 | ,003 |
| M | 1,546 | 1 | 1,546 | 2,755 | ,110 |
| ZYMV | 7,040 | 1 | 7,040 | 12,548 | ,002 |
| Whey App. * M | ,677 | 2 | ,338 | ,603 | ,555 |
| Whey App. * ZYMV | 3,241 | 2 | 1,620 | 2,888 | ,075 |
| M * ZYMV | 2,122 | 1 | 2,122 | 3,782 | ,064 |
| Whey App. * M * ZYMV | 1,252 | 2 | ,626 | 1,116 | ,344 |
| Error | 13,466 | 24 | ,561 |  |  |
| Total | 560,677 | 36 |  |  |  |
| Corrected Total | 37,487 | 35 |  |  |  |
| a. R Squared = ,641 (Adjusted R Squared = ,476) | | | | | |

| **Tests of Between-Subjects Effects** | | | | | |
| --- | --- | --- | --- | --- | --- |
| Dependent Variable: AMF Spore Number | | | | | |
| Source | Type III Sum of Squares | df | Mean Square | F | Sig. |
| Corrected Model | 369284,400^a^ | 5 | 73856,880 | 27,652 | ,000 |
| Intercept | 5347585,200 | 1 | 5347585,200 | 2002,141 | ,000 |
| Whey App. | 146607,200 | 2 | 73303,600 | 27,445 | ,000 |
| M | ,000 | 0 | . | . | . |
| ZYMV | 152368,133 | 1 | 152368,133 | 57,047 | ,000 |
| Whey App. * M | ,000 | 0 | . | . | . |
| M * ZYMV | ,000 | 0 | . | . | . |
| Whey App. * ZYMV | 70309,067 | 2 | 35154,533 | 13,162 | ,000 |
| Whey App. * M * ZYMV | ,000 | 0 | . | . | . |
| Error | 64102,400 | 24 | 2670,933 |  |  |
| Total | 5780972,000 | 30 |  |  |  |
| Corrected Total | 433386,800 | 29 |  |  |  |
| a. R Squared = ,852 (Adjusted R Squared = ,821) | | | | | |

| **Tests of Between-Subjects Effects** | | | | | |
| --- | --- | --- | --- | --- | --- |
| Dependent Variable: Mycorrhizal Intensity | | | | | |
| Source | Type III Sum of Squares | df | Mean Square | F | Sig. |
| Corrected Model | 31,684^a^ | 5 | 6,337 | 3,064 | ,028 |
| Intercept | 1264,122 | 1 | 1264,122 | 611,232 | ,000 |
| Whey App. | 26,194 | 2 | 13,097 | 6,333 | ,006 |
| M | ,000 | 0 | . | . | . |
| ZYMV | ,622 | 1 | ,622 | ,301 | ,588 |
| Whey App. * M | ,000 | 0 | . | . | . |
| M * ZYMV | ,000 | 0 | . | . | . |
| Whey App. * ZYMV | 4,868 | 2 | 2,434 | 1,177 | ,325 |
| Whey App. * M * ZYMV | ,000 | 0 | . | . | . |
| Error | 49,636 | 24 | 2,068 |  |  |
| Total | 1345,442 | 30 |  |  |  |
| Corrected Total | 81,320 | 29 |  |  |  |
| a. R Squared = ,390 (Adjusted R Squared = ,262) | | | | | |

| **Tests of Between-Subjects Effects** | | | | | |
| --- | --- | --- | --- | --- | --- |
| Dependent Variable: Mycorrhizal Frequency | | | | | |
| Source | Type III Sum of Squares | df | Mean Square | F | Sig. |
| Corrected Model | 181,167^a^ | 5 | 36,233 | ,629 | ,680 |
| Intercept | 257200,206 | 1 | 257200,206 | 4462,714 | ,000 |
| Whey App. | 8,229 | 2 | 4,114 | ,071 | ,931 |
| M | ,000 | 0 | . | . | . |
| ZYMV | 148,252 | 1 | 148,252 | 2,572 | ,122 |
| Whey App. * M | ,000 | 0 | . | . | . |
| M * ZYMV | ,000 | 0 | . | . | . |
| Whey App. * ZYMV | 24,686 | 2 | 12,343 | ,214 | ,809 |
| Whey App. * M * ZYMV | ,000 | 0 | . | . | . |
| Error | 1383,195 | 24 | 57,633 |  |  |
| Total | 258764,568 | 30 |  |  |  |
| Corrected Total | 1564,362 | 29 |  |  |  |
| a. R Squared = ,116 (Adjusted R Squared = -,068) | | | | | |

| **Tests of Between-Subjects Effects** | | | | | |
| --- | --- | --- | --- | --- | --- |
| Dependent Variable: Disease Severity | | | | | |
| Source | Type III Sum of Squares | df | Mean Square | F | Sig. |
| Corrected Model | 314,674^a^ | 5 | 62,935 | 4,736 | ,004 |
| Intercept | 159767,816 | 1 | 159767,816 | 12022,410 | ,000 |
| Whey App. | 196,209 | 2 | 98,104 | 7,382 | ,003 |
| M | 42,960 | 1 | 42,960 | 3,233 | ,085 |
| ZYMV | ,000 | 0 | . | . | . |
| Whey App. * M | 75,505 | 2 | 37,752 | 2,841 | ,078 |
| Whey App. * ZYMV | ,000 | 0 | . | . | . |
| M * ZYMV | ,000 | 0 | . | . | . |
| Whey App. * M * ZYMV | ,000 | 0 | . | . | . |
| Error | 318,940 | 24 | 13,289 |  |  |
| Total | 160401,430 | 30 |  |  |  |
| Corrected Total | 633,614 | 29 |  |  |  |
| a. R Squared = ,497 (Adjusted R Squared = ,392) | | | | | |
